# Nanoparticles based on the zwitterionic pillar[5]arene and Ag^+^: synthesis, self-assembly and cytotoxicity in the human lung cancer cell line A549

**DOI:** 10.3762/bjnano.11.33

**Published:** 2020-03-05

**Authors:** Dmitriy N Shurpik, Denis A Sevastyanov, Pavel V Zelenikhin, Pavel L Padnya, Vladimir G Evtugyn, Yuriy N Osin, Ivan I Stoikov

**Affiliations:** 1 Kazan Federal University, A.M. Butlerov Chemistry Institute, 420008 Kremlevskaya, 18, Kazan, Russian Federation; 2 Institute of Fundamental Medicine and Biology, Kazan Federal University, 420008 Kremlevskaya, 18, Kazan, Russian Federation; 3 Interdisciplinary Centre for Analytical Microscopy, Kazan Federal University, 420008 Kazan, Kremlevskaya 18, Russian Federation

**Keywords:** cytotoxicity, macrocyclic receptors, nanoparticles, pillar[5]arene, self-assembly, silver

## Abstract

For the first time, stable pillar[5]arene/Ag^+^ nanoparticles, consisting of water-soluble pillar[5]arene containing γ-sulfobetaine fragments and Ag^+^ ions without Ag–Ag bonds, were synthesized and characterized. The pillar[5]arene/Ag^+^ (ratio 1:10) nanoparticles obtained were cubic with a rib length of 100 nm and are less cytotoxic than Ag^+^ ions. The survival of the A549 model cells in the presence of pillar[5]arene/Ag^+^ (1:10) nanoparticles at a concentration of 30 and 40 μM was 76% and 55%, while in the absence of pillar[5]arene, the cell survival for free Ag^+^ ions at the same concentration was 30% and 10%, respectively. The results can be used to create new antibacterial materials and 2D biomedical coatings.

## Introduction

The first use of silver (Ag) as a medicine dates back to Hippocrates, who used it as an antibacterial agent for the treatment of ulcers [1]. Over the past thousands of years, the use of this metal has grown into an entire industry [1]. A separate area of this industry is the use of silver nanoparticles (AgNPs), which are the source of Ag^+^ ions in many commercial products, such as food packaging, odor-resistant fabrics, household appliances and medical devices [2–4]. Silver is well known for its antimicrobial activity, and Ag^+^ ion is usually considered a biologically active substance [5–8]. However, it is known that the effect of Ag^+^ on the human body is toxic and can cause diseases such as argyria (irreversible staining of the skin in gray color) [5–8]. The European Commission Scientific Committee on Emerging and Newly Identified Health Risks (SCENIHR) has concluded that AgNPs may have toxicological properties different from the main substance, but their risks should be evaluated on a case-by-case basis [7].

In order to reduce the toxicity of Ag^+^ ions, multicore complexes prepared on the basis of macrocyclic ligands [9–13] and silver ions are of greatest interest. The ability of macrocyclic systems to form supramolecular associates, due to noncovalent interactions with Ag^+^, can reduce toxicity and preserve the biomedical properties of the resulting nanoscale particles that do not contain Ag–Ag bonds [14]. However, the main problem is the fact that macrocyclic hosts have low solubility in water, which significantly narrows their application [15–20]. Therefore, we propose to use water-soluble derivatives of decasubstituted pillar[5]arenes containing sulfobetaine fragments in their structure as target structures. It was shown that the introduction of such zwitterionic fragments into the structure leads to toxicity reduction [21], an increase in the solubility of substances in water [22] and ensures the stability of supramolecular assemblies to pH changes [22–23]. Another remarkable property of sulfobetaine fragments is the ability to interact with silver ions [24–26] and participate in the stabilization of quantum dots [27]. It is worth noting that the use of polymer systems containing sulfobetaine fragments to produce nanoparticles without Ag–Ag bonds leads to the formation of a limited number of particle forms [28]. Macrocycle molecules do not swell and have a greater degree of freedom in aqueous solutions while zwitterionic fragments prevent their aggregation. The lack of aggregation and presence of a preorganized macrocyclic structure makes the process of controlled supramolecular self-assembly possible with certain coordination sites. In this paper, we report the first example of using nontoxic, water-soluble pillar[5]arene derivatives containing sulfobetaine fragments to produce nanoparticles with Ag^+^ ions using controlled supramolecular self-assembly.

## Results and Discussion

### Synthesis of decasubstituted pillar[5]arenes containing sulfobetaine fragments

**Figure 1:**
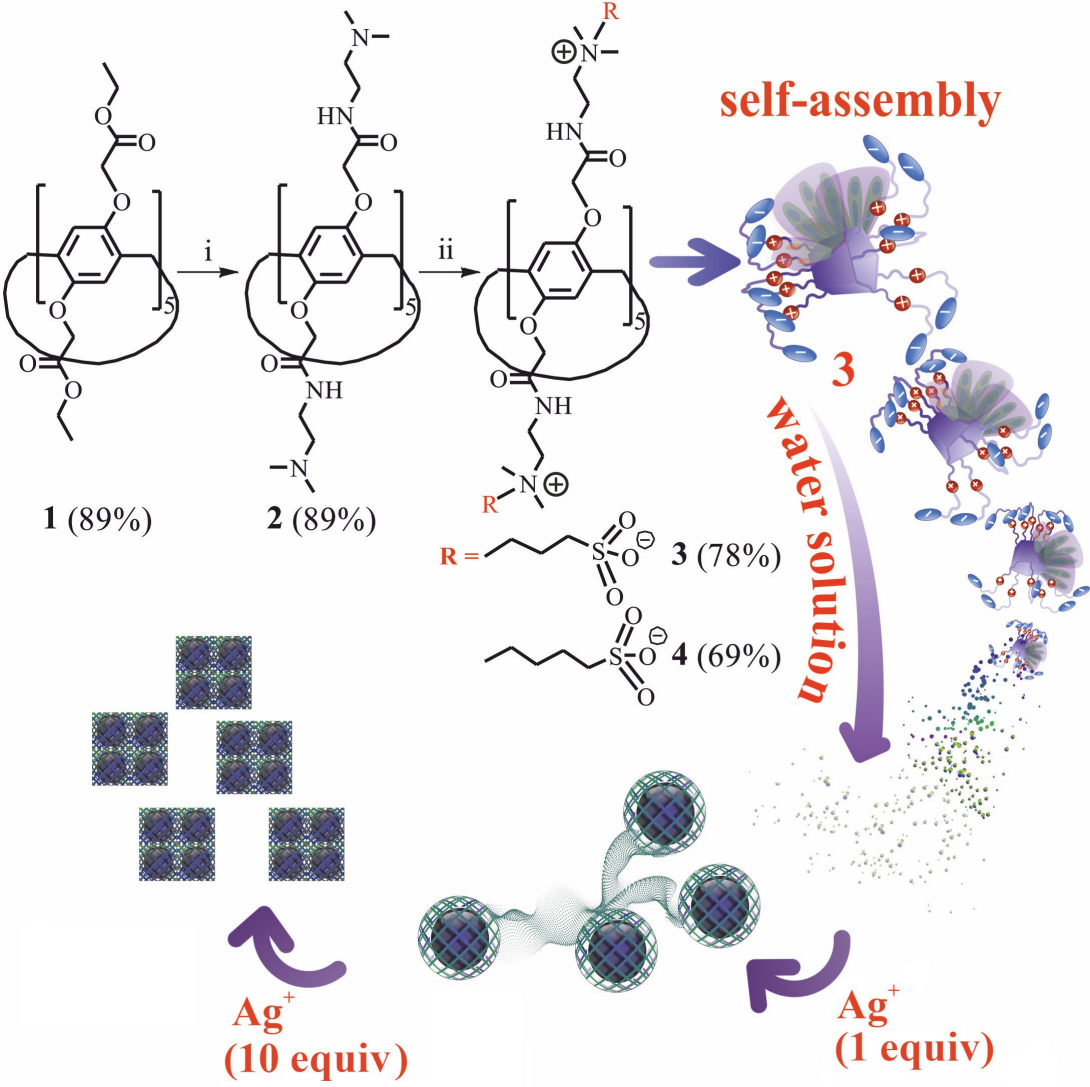
Synthesis of the macrocycles **3** and **4**. The sketch represents self-organization of the **3**/Ag^+^ system depending on the amount of silver. Reagents and conditions: i – *N,N*-dimethylethylenediamine, MeOH, reflux; ii – 1,3-propanesultone or 1,4-butanesultone, DMF, 100 °C.

We previously developed a method [29] for introducing tertiary amino groups into the structure of pillar[5]arene. Diamines containing both primary and tertiary amino groups were used as aminating agents [30]. In this work, we used commercially available *N*,*N*-dimethylethylenediamine, because there are no side reactions due to the presence of two methyl groups, and the process of further quaternization proceeds under mild conditions [31]. The macrocycle **2** with 89% yield was obtained by aminolysis of decaether **1** by *N*,*N*-dimethylethanediamine (Figure 1, Supporting Information File 1, Figures S1–S12).

In order to introduce sulfobetaine fragments into the pillar[5]arene structure, we used 1,3-propane- and 1,4-butanesultones. Sultones are convenient precursors that make it possible to introduce sulphonate fragments under mild conditions into the macrocycle structure by breaking the ester bond of the γ- and δ-sultone cycle under the action of a nucleophilic agent [32–33]. As a result, the target products **3** and **4** were obtained by reaction of macrocycle **2** with 1,3-propane- and 1,4-butanesultone in DMF at 100 °C for 72 hours with yields of 78% and 69% respectively (Figure 1, Supporting Information File 1, Figures S1–S12). The structure and composition of the obtained compounds were characterized by a number of physical methods, i.e., ^1^H and ^13^С NMR spectroscopy, two-dimensional ^1^H,^1^H-NOESY NMR spectroscopy, IR spectroscopy, MALDI–TOF mass-spectrometry and elemental analysis.

### Association of decasubstituted pillar[5]arenes with Ag^+^

In addition, the self-association of the obtained macrocycles **2**–**4** and their aggregation with AgNO_3_ in water was studied (Figure 1). The study of self-association of macrocycles **2**–**4** was carried out in water using the dynamic light scattering (DLS) method. It turned out that the decaamine **2**, due to its poor solubility in water, does not form any self-associates across the entire range of concentrations studied (10^−3^–10^−5^ M). However, the introduction of ten sulfobetaine fragments in the case of compounds **3** and **4** contributes to the solubility of pillar[5]arenes in water. It was shown that macrocycles **3** and **4** do not form stable self-associates in the studied concentration range (10^−3^–10^−5^ M) in water.

The next step was the study of the interaction of pillar[5]arenes **3** and **4** with AgNO_3_ by DLS, UV–vis and ^1^H, ^13^C, 2D NMR spectroscopy. It was shown by UV–vis spectroscopy that addition of an excess of AgNO_3_ to macrocycles **3** and **4** does not lead to an absorption maximum shift. It is worth noting that, in the case of macrocycle **3**, the baseline is raised (Supporting Information File 1, Figure S13). In the case of the compound **4** in the UV–vis spectra the baseline rise was not observed. Apparently, the baseline rise in the UV–vis spectra is due to the processes of Ag^+^ aggregation with pillar[5]arene **3**.

**Figure 2:**
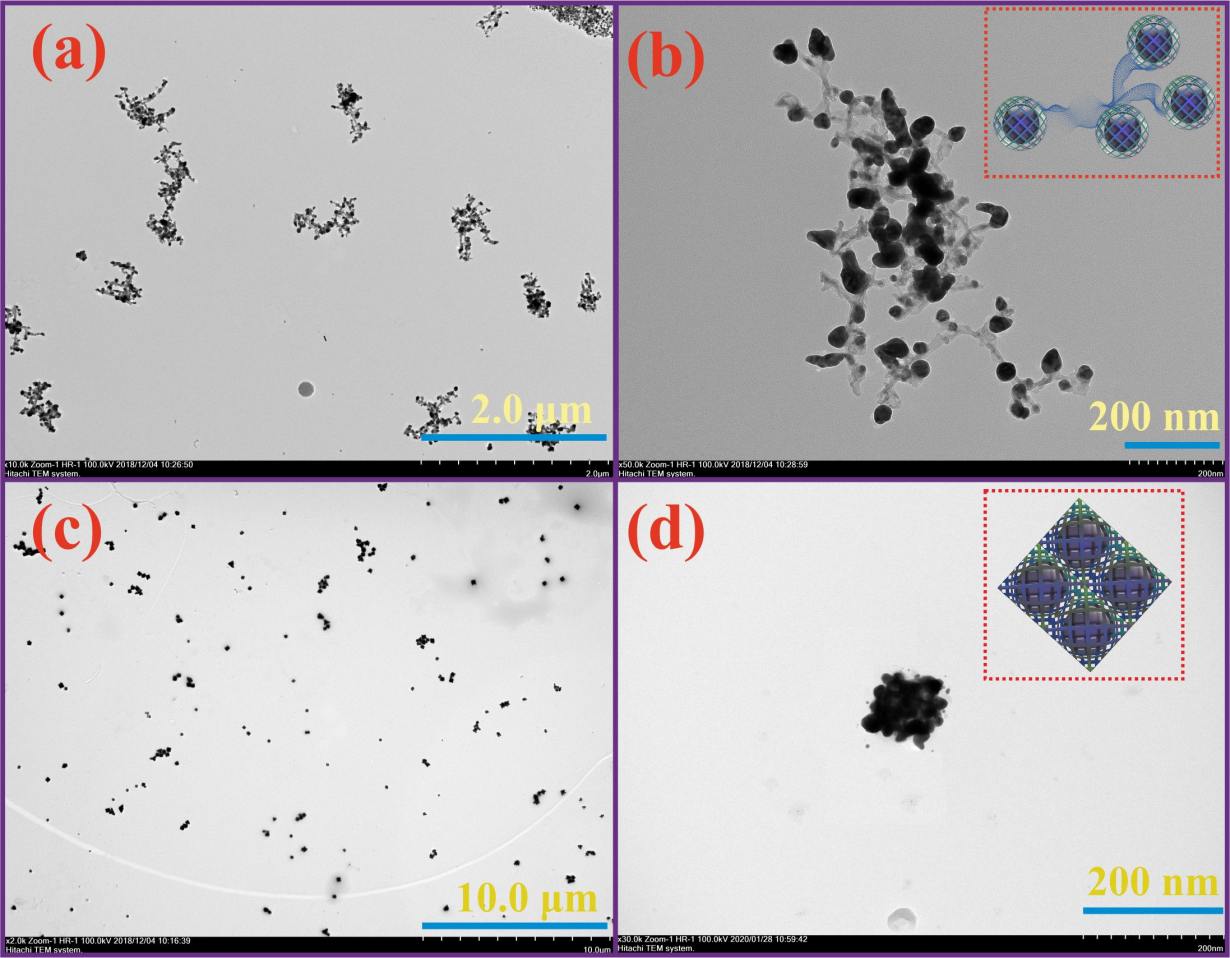
TEM images of pillar[5]arene **3**/Ag^+^: (a) and (b) 1:1 associates in water (10^−4^ M); (c) and (d) 1:10 associates in water (*c*(**3**) = 10^−4^ М, *c*(AgNO_3_) = 10^−3^ М).

To confirm the hypothesis about the use of water-soluble pillar[5]arenes capable of forming nontoxic, nanoscale associates with Ag^+^, we used the DLS method and study the macrocycle/AgNO_3_ systems in a ratio of 2:1, 1:1, 1: 5, 1:10, and 1:15 over a range of concentrations (10^−3^–10^−5^ M). It is interesting to note that the stable monodisperse **3**/Ag^+^ associates were formed only in the case of macrocycle **3** (10^−3^–10^−5^ M) in the presence of Ag^+^ (Supporting Information File 1, Table S1) over a wide range of concentrations. Thus, the formation of monodisperse nanometer-sized associates occurs with a ten-fold excess of Ag^+^ (10^−2^ M) at a macrocycle **3** concentration of 10^−3^ M (Supporting Information File 1, Table S1). With a decrease in the concentration of **3** (10^−4^–10^−5^ M), the formation of monodisperse nanometer-sized associates occurs over a wider range of concentrations of Ag^+^ (10^−3^–10^−5^ M) (Supporting Information File 1, Table S1). The minimum polydispersity index (PDI) values (PDI = 0.041) were obtained for the macrocycle **3**/AgNO_3_ = 1:10 ratio (*c*(**3**) = 10^−4^ M, *c*(AgNO_3_) = 10^−3^ M) with an average hydrodynamic diameter of 122 nm (Supporting Information File 1, Figure S14). The high value of the zeta potential of the system **3**/Ag^+^ = 1:10 (ζ = +31.8 mV) additionally confirm the formation of a stabilized supramolecular system (Supporting Information File 1, Figure S15). According to the TEM data, the **3**/Ag^+^ associates (10^−4^ M) in a 1:1 ratio are nanoparticles consisting of spherical 0D (*d* = 40 nm) and elongated 1D (*d* = 15 nm) structures (Figure 2a,b, Supporting Information File 1, Figures S16–S20). However, with an increase of the Ag^+^ concentration (10^−3^ M), the **3**/Ag^+^ associates are characterized by a cubic shape with a rib length of 100 nm (Figure 2c,d, Supporting Information File 1, Figures S16–S20). In the case of the **4**/Ag^+^ system, unstable polydisperse systems are formed in all the studied concentration ranges (Supporting Information File 1, Table S2). Apparently, this is due to the structure of macrocycles **3** and **4**, namely, the increase in the length of the linker between ammonium and sulfo groups, as well as the spatial structure of the **3**/Ag^+^ and **4**/Ag^+^ associates. It should be noted that the formation of a reduced form of silver Ag(0) was not recorded under all studied conditions. This fact was confirmed by the absence of an absorption band corresponding to Ag–Ag bonds in AgNPs in the UV–vis spectra [10]. Thus, studies of the **3**/Ag^+^ association show the formation of time-stable nanoparticles of macrocycle **3**/Ag^+^ not containing Ag–Ag bonds.

**Figure 3:**
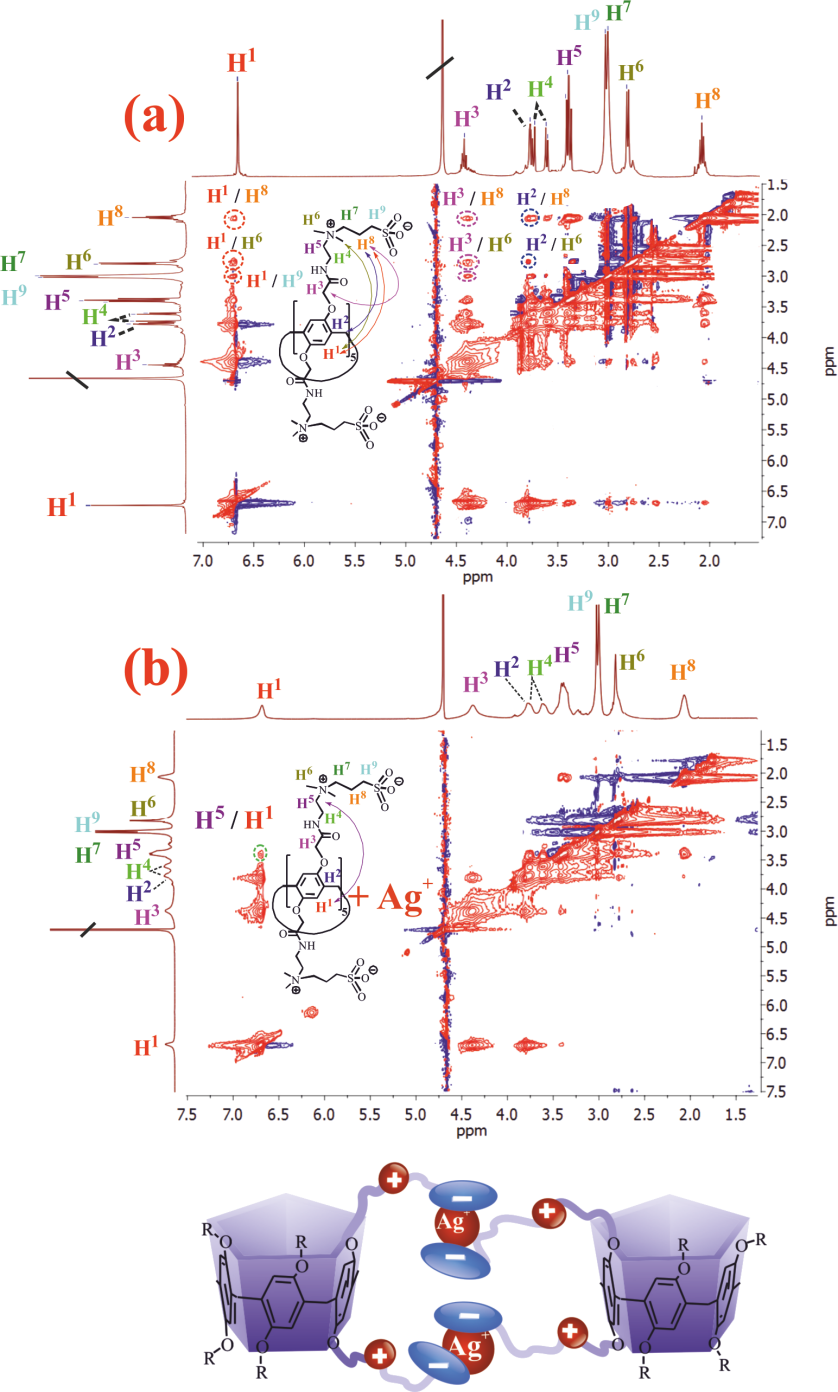
2D ^1^Н,^1^Н-NOESY NMR spectra: (a) macrocycle **3** (10^−3^ M); (b) associate **3**/Ag^+^ at a ratio of 1:10 in D_2_O at 298 K and the estimated structure of the complex.

To confirm this hypothesis, we studied the interaction of macrocycles **3** and **4** with AgNO_3_ by ^1^Н NMR spectroscopy. The analysis of the experimental data obtained by ^1^Н NMR spectroscopy unfortunately did not allow us to determine the nature of the interaction by changing the position of the host and guest chemical shifts. The changes that were recorded in the ^1^H NMR spectrum of the **3**/Ag^+^ system (1:1, 10^−3^ M) were related to the broadening of the macrocycle signals, which indirectly indicates the aggregation process and the formation of **3**/Ag^+^ associates [34–36]. Therefore, we used the 2D ^1^H,^1^H-nuclear Overhauser effect spectroscopy (NOESY) NMR methods (Figure 3), 2D ^1^H,^1^H-rotational frame nuclear Overhauser effect spectroscopy (ROESY) NMR and 2D diffusion ordered spectroscopy (DOSY) NMR methods to confirm the formation of associates. There are observed cross peaks between propylene protons of sulfobetaine fragment **H^8^** and ArH protons **H^1^** pillar[5]arene, cross peaks between protons of sulfobetaine fragment **H^8^** and the protons of the methylene bridge **H^2^**, as well as cross peaks between the protons of the methyl groups of the ammonium fragment **H^6^** and the protons of the methylene bridge **H^2^**, and cross peaks between the protons of the sulfobetaine fragment **H^8^** and the hydroxymethylene protons **H^3^** in the case of macrocycle **3** in the 2D ^1^H,^1^H-NOESY NMR spectrum (Figure 3a). In the case of macrocycle **4** in the 2D ^1^H,^1^H-NOESY NMR spectrum, similar cross peaks are observed between the protons of the butylidene fragment and the protons of ArH and the methylene bridge (Supporting Information File 1, Figure S21). The presence of these cross peaks indicates a spatially close location of sulfobetaine and aromatic fragments of pillar[5]arenes **3** and **4**.

It is worth noting that for the mixture of **3**/Ag^+^ in a ratio of 1:10 (*c*(**3**) = 10^−3^ M, *c*(AgNO_3_) = 10^−2^ M) in D_2_O (Figure 3b) cross peaks **Н^8^**/**Н^1^**, **Н^8^**/**Н^2^**, **Н^6^**/**Н^2^**, **Н^8^**/**Н^3^** (characterizing the spatial structure of **3**) are not observed in the 2D ^1^H,^1^H-NOESY NMR spectrum. However, the addition of Ag^+^ excess to macrocycle **4** does not lead to a significant change in the 2D NOESY NMR spectrum. This is apparently due to a change in the spatial structure of macrocycle **3** and the formation of the **3**/Ag^+^ associate, which is not observed in the case of macrocycle **4**. The obtained results are in good agreement and confirmed by the 2D ^1^H,^1^H-ROESY NMR spectroscopy. 2D ROESY NMR spectroscopy is one of the most effective tools for the study of inter- and intramolecular interactions of complex systems. The presence of cross peaks generated by nuclear Overhauser effects (NOE) between spatially close protons gives useful information about the dynamics and averaged relative inter/intramolecular proton distances of these particles within 0.4 nm in solution [37–39]. **Н^8^**/**Н^1^**, **Н^8^**/**Н^2^**, **Н^6^**/**Н^2^**, **Н^8^**/**Н^3^** cross peaks are observed in the 2D ^1^H,^1^H-ROESY NMR spectrum of **3**, which confirms the hypothesis of a spatially close arrangement of sulfobetaine and aromatic fragments of pillar[5]arene **3** (Supporting Information File 1, Figure S22). However, when AgNO_3_ was added to the macrocycle **3** (Supporting Information File 1, Figure S23), these cross peaks disappeared, which indicated an increase in the distance (>0.4 nm) between the bound NOE protons. Thus, the analysis of experimental data indicates that Ag^+^ cation is located outside of the macrocycle **3**, between the aromatic ring and substituent fragments, forming supramolecular structures that do not contain a Ag–Ag bond. This hypothesis is in good agreement with the literature data [40–41].

**Figure 4:**
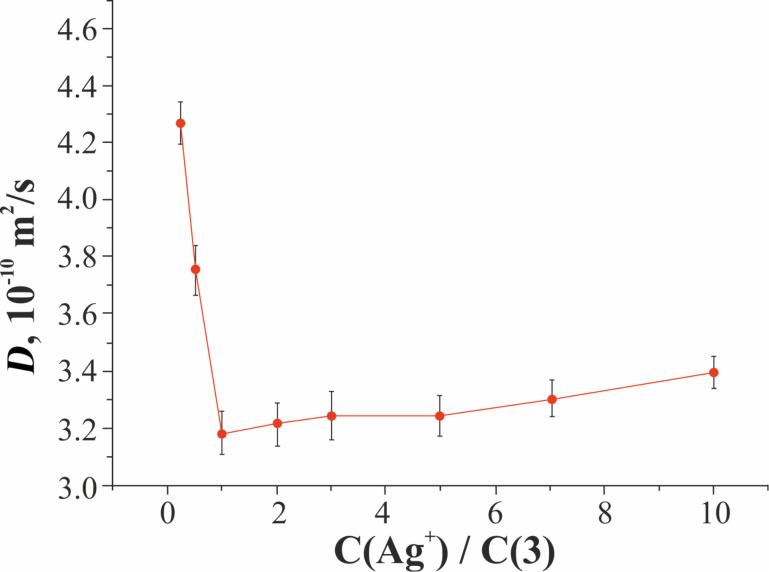
Change in the diffusion coefficients of macrocycle **3** and **3**/Ag^+^ associates.

The formation of the **3**/Ag^+^ associate was additionally confirmed by two-dimensional DOSY NMR spectroscopy. The diffusion coefficients of **3** and **3**/Ag^+^ at 298 K (10^–3^ M) were determined (Figure 4, Supporting Information File 1, Table S3). Figure 4 shows that the diffusion coefficients for pillar[5]arene **3** and **3**/Ag^+^ nanoparticles are different. The self-diffusion coefficient of pillar[5]arene **3** is greater (*D* = 4.22 × 10^–10^ m^2^ s^−1^), which indicates its greater mobility in solution in comparison with **3**/Ag^+^ associates. This is consistent with the fact that the molecular weight of **3**/Ag^+^ is greater than the molecular weight of pillar[5]arene **3**. When a small amount of Ag^+^ (*c*(Ag^+^)/*c*(**3**) = 0.5) is added to the pillar[5]arene **3** solution, its self-diffusion coefficient sharply decreases (*D* = 3.75 × 10^−10^ m^2^ s^−1^), which indicates a decrease in mobility pillar[5]arene due to the formation of the associate. The minimum diffusion coefficient is observed at *c*(Ag^+^)/*c*(**3**) = 1 (*D* = 3.19 × 10^−10^ m^2^ s^−1^). With an increase in the Ag^+^ concentration, the self-diffusion coefficients equalize (Figure 4).

With a further increase in the Ag^+^ concentration, the self-diffusion coefficient of pillar[5]arene begins to increase to the ratio *c*(Ag^+^)/*c*(**3**) = 10 (*D* = 3.40 × 10^−10^ m^2^ s^−1^), while a further increase in the Ag^+^ concentration does not lead to a change in the self-diffusion coefficient of pillar[5]arene **3**.

In isotropic solutions, diffusion is closely related to the size of the molecules in accordance with the Stokes–Einstein equation:


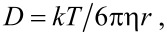


where *r* is the hydrodynamic radius of the studied molecule. Therefore, quantitative measurements of the effective diffusion provide information on the size and interactions of a diffusing molecule or supramolecular complex [42]. The tendency for the formation of nanoparticles in the system pillar[5]arene **3** and Ag^+^ with increasing Ag^+^ concentration was additionally confirmed by the hydrodynamic radius of the **3**/Ag^+^ associates calculated using the Stokes–Einstein equation (Supporting Information File 1, Figure S24). When passing from the ratio *c*(Ag^+^)/*c*(**3**) = 0–1, an increase in *r* (0.45–0.91 nm) occurs, then a gradual decrease in *r* (0.91–0.70 nm) to the value *c*(Ag^+^)/*c*(**3**) = 10, after which *r* does not change. The differences in sizes obtained by DLS and DOSY can be explained by the low sensitivity of the NMR spectroscopy method. The DLS method is a more sensitive method and captures the entire range of particles in solution, and the DOSY NMR spectroscopy method is less sensitive and captures only small particles formed during the association process [43]. Previously, the ability of pillar[5]arenes to interact with various anions [11] was shown. In this regard, to assess the possible interfering effect on the formation of **3**/Ag^+^ associates, the anions NO_3_^−^, СH_3_COO^−^, and SO_4_^2−^ present in the solution were studied, and their possible competition with Ag^+^ for the pillar[5]arene **3** was also studied. However, the formation of macrocycle **3** complexes with the indicated anions was not detected by UV–vis, NMR spectroscopy, which indicates the absence of their interfering effect.

### Cytotoxicity of synthesized macrocycles and associates of macrocycle/Ag^+^

**Figure 5:**
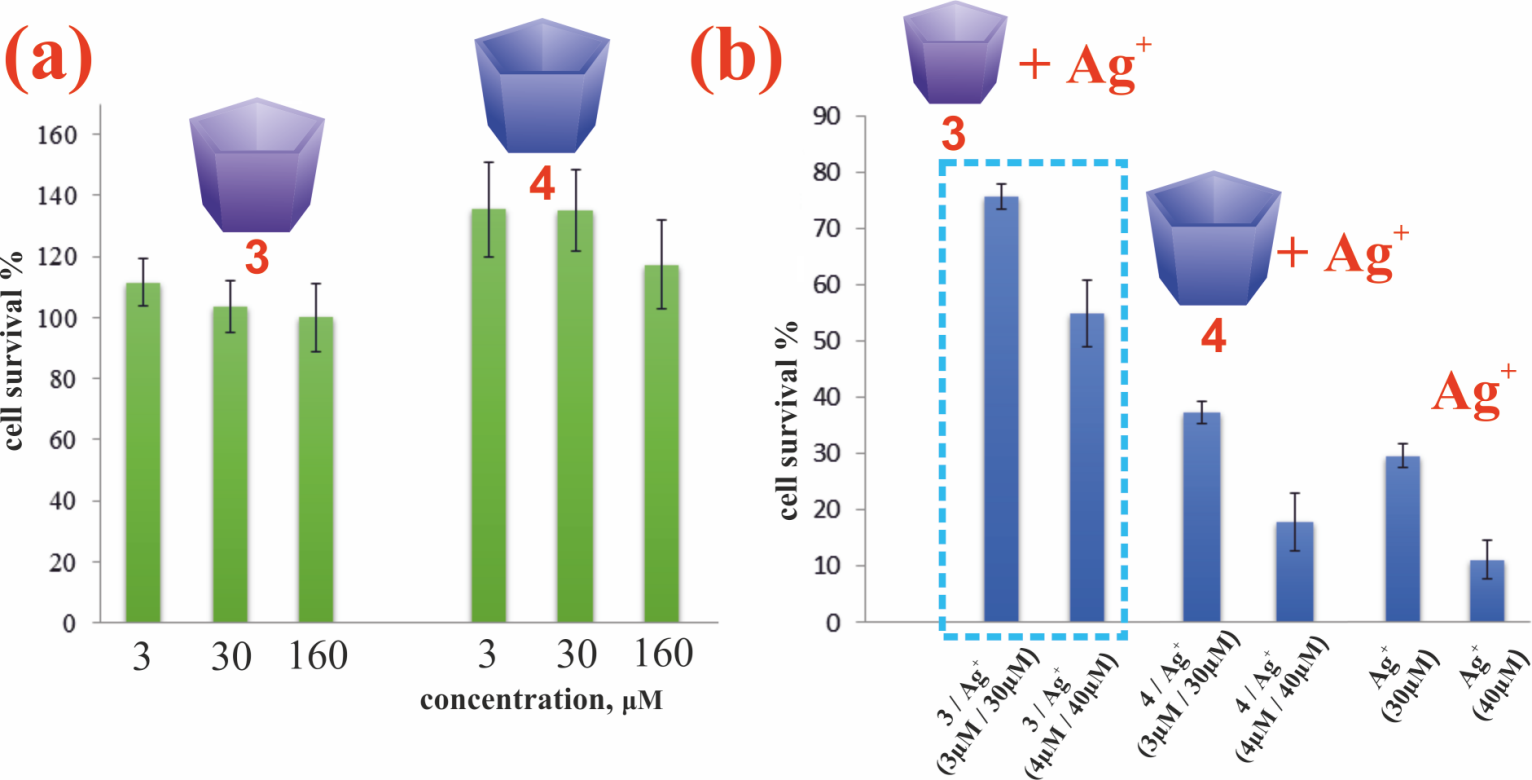
(a) Evaluation of the effect of pillar[5]arenes **3** and **4** on the viability of A549 cells (*p* ≤ 0.05); (b) Evaluation of the effect of associates of **3**/Ag^+^ on the viability of A549 cells (*p* ≤ 0.05).

It was previously shown that water-soluble pillar[5]arenes did not have a pronounced toxic effect in the range of studied concentrations of 10–500 μg/mL [44–46]. In order to study the cytotoxic properties of the obtained **3**/Ag^+^ nanoparticles, we evaluated the cytotoxicity of the synthesized water-soluble pillar[5]arenes **3** and **4**. The results indicate that macrocycles **3** and **4** did not show statistically significant cytotoxic activity against A549 cells over the range of studied concentrations (3–160 μM) (Figure 5a). Characterization of changes in the viability of A549 cells under the action of the pillar[5]arenes was performed by the MTT test [47].

Since in the case of macrocycle **3**, in the presence of an excess of AgNO_3_, stable nanoparticles are formed, we further studied the ability of the **3**/Ag^+^ associates to reduce the toxicity of Ag^+^ for eukaryotic cells. We first carried out the calculation of the minimum inhibitory concentration for silver ions. The minimum inhibitory concentration of silver ions for A549 cells was 28.4 μM. The IC_50_ Ag^+^ value, determined by us for A549 cells, is consistent with the literature data [7], where it was shown that the value of this indicator for eukaryotic cells is in the range of 9–37 μM. Based on the results obtained, silver ion concentrations of 30 and 40 μM were used for further work to compare the toxicity of the **3**/Ag^+^ nanoparticles and Ag^+^.

The viability of A549 cells in the presence of **3**/Ag^+^ nanoparticles (*c*(**3**) = 3 μM, *c*(AgNO_3_) = 30 μM) was significantly higher than the values of this indicator when processing only Ag^+^ and **4**/Ag^+^ (*c*(**4**) = 3 μM, *c*(AgNO_3_) = 30 μM) (Figure 5b). The value of this indicator was 76% at a concentration of Ag^+^ of 30 μM. In the Ag^+^ monoprocessing variant, the A549 cell survival rate was 30%. When Ag^+^ was used at a concentration of 4 µg/mL, the cell survival after 24 h of incubation was only 11% (Figure 5b). Adding **3**/Ag^+^ nanoparticles to the system allowed us to increase the value of this indicator to 55%, respectively. The results reliably indicate a decrease in the toxicity of **3**/Ag^+^ nanoparticles compared with Ag^+^ for eukaryotic cells.

## Conclusion

Thus, for the first time, water-soluble decasubstituted pillar[5]arenes, containing γ- and δ-sulfobetaine fragments were synthesized. It was shown by the DLS method that the obtained pillar[5]arenes **3** and **4** did not form stable self-associates in aqueous solution. The association of macrocycle **3** with AgNO_3_ was confirmed by DLS, UV–vis and 2D NMR spectroscopy methods. As a result of the association, monodisperse **3**/Ag^+^ nanoparticles are formed in the entire wide range of concentrations studied (10^−3^–10^−5^M). The minimum PDI values (0.041) were obtained for the macrocycle/AgNO_3_ = 1:10 ratio (*c*(**3**) = 10^−4^ M, *c*(AgNO_3_) = 10^−3^ M) with an average hydrodynamic diameter of 122 nm. The high values of the zeta potential of the system ζ (**3**/Ag^+^) = +31.8 mV additionally confirm the formation of a stabilized supramolecular system. The cytotoxic effect of pillar[5]arenes **3** and **4** on A549 cells in the MTT test was characterized. The compounds did not show a statistically significant cytotoxic activity against A549 cells in the range of concentrations studied (3–160 μM). **3**/Ag^+^ nanoparticles had lower cytotoxicity than Ag^+^ ions. Therefore, it was concluded that macrocycle **3** has the ability to reduce the toxicity of Ag^+^. The survival rate of A549 model cells was 76% for a concentration of silver ions of 30 μM and 55% for a concentration of silver ions of 40 μM. The results can be used to create new antibacterial materials and 2D biomedical coatings.

## Experimental

^1^H NMR, ^13^C and 2D NOESY NMR spectra were obtained on a Bruker Avance-400 spectrometer (^13^С{^1^H} – 100 MHz and ^1^H and 2D NOESY, ROESY – 400 MHz). The chemical shifts were determined against the signals of residual protons of deuterated solvent (DMSO-*d*_6_, D_2_O). The 2D ^1^H,^1^H-ROESY spectra were recorded using the roesyphpr standard pulse sequence at 298 K. The mixing time was 500 ms. The concentration of sample solutions was 3–5%. The attenuated total internal reflectance IR spectra were recorded with a Spectrum 400 (Perkin Elmer) Fourier spectrometer. Elemental analysis was performed with a Perkin Elmer 2400 Series II instrument. Mass spectra (MALDI-TOF) were recorded on an Ultraflex III mass spectrometer in a 4-nitroaniline matrix. The melting points were determined using a Boetius Block apparatus. Additional control of the purity of compounds and monitoring of the reaction were carried out by thin-layer chromatography using Silica G, 200 µm plates, UV 254. Most chemicals were purchased from Aldrich and used as received without additional purification. The organic solvents were purified in accordance with standard procedures. Pillar[5]arene **1** was synthesized according to the literature procedures [48].

### Synthesis of compounds **2**–**4**

#### 4,8,14,18,23,26,28,31,32,35-deca-[*N*-(2’,2’-dimethylaminoethyl)carbamoylmethoxy]pillar[5]arene (**2**)

Pillar[5]arene **1** (0.30 g, 0.2 mmol) was suspended in methanol (10 mL). Then *N,N*-dimethylethan-1,2-diamine (0.26 g, 3.0 mmol, 0.33 mL) was added to the reaction mixture. The reaction mixture was refluxed for 72 h. The residue was dissolved in chloroform (20 mL) and washed several times with distilled water. The organic layer was separated and dried (molecular sieves, 3 Å), and the solvent was removed under reduced pressure. The residue was dried under reduced pressure for 30 min and a light-yellow viscous oil was obtained. Yield 0.34 g (89%). ^1^H NMR (400 MHz, DMSO-*d*_6_, 298 K) δ (ppm): 2.08 (s, 60H, -CH_3_), 2.25 (t, 20H, ^3^*J*_HH_ = 6.6 Hz, -NH-CH_2_-C*H*_2_-N(CH_3_)_2_), 3.21 (m, 20H, -NH-C*H*_2_-CH_2_-N(CH_3_)_2_), 3.79 (s, 10H, -CH_2_-), 4.34 (br s, 20H, -O-C*H*_2_-C(O)-NH-), 6.84 (s, 10H, ArH), 7.83 (10H, br t, -NH-); ^13^С NMR (100 MHz, DMSO-*d*_6_, 298 K) δ (ppm): 28.80, 36.59, 46.47, 51.37, 67.71, 114.71, 127.97, 148.95, 167.64; IR (ν/cm^−1^): 2967, 2932, 2812 (-CH_2_-, -CH=), 3311 (N-H), 1661 (С=О), 1201 (Ar-O-CH_2_-); MALDI–TOF MS *m*/*z*: calcd for [M^+^], 1892.1; found for [M + Na]^+^, 1915.4; Anal. calcd for C_95_H_150_N_20_O_20_: C, 60.30; H, 7.99; N, 14.80; found: C, 60.01; H, 7.58; N, 14.63.

### General procedure for the synthesis of compounds **3** and **4**

Pillar[5]arene **2** (0.3g, 0.16 mmol) was dissolved in 15 mL of anhydrous DMF. Then 1,3-propanesultone or 1,4-butanesultone (4.7 mmol) was added to the reaction mixture. The reaction mixture was stirred at a temperature of 100 °C under argon atmosphere for 48 h. The reaction mixture was cooled and poured into 20 mL of diethyl ether. The resulting yellow powder was collected by filtration. The crude product was recrystallized from acetone/water 10:3 (20 mL). The resulting light yellow powder was dried under reduced pressure.

#### 4,8,14,18,23,26,28,31,32,35-deca-[*N*-(2',2'-dimethyl-2'-(3''-sulfonatopropyl)ammoniummethyl)carbamoylmethoxy]pillar[5]arene (**3**)

Yield 0.29 g (78%). ^1^H NMR (400 MHz, D_2_O, 298 K) δ (ppm): 2.20 (m, 20H, -CH_2_-C*H*_2_-CH_2_-SO_3_^−^), 2.94 (m, 60H, -N^+^(C*H*_3_)_2_-), 3.14 (m, 40H, -N^+^(CH_3_)_2_- C*H*_2_-CH_2_-C*H*_2_-SO_3_^−^), 3.54 (m, 20H, -NH-CH_2_-C*H*_2_-N^+^(CH_3_)_2_-), 3.74–3.90 (m, 20H, -NH-C*H*_2_-CH_2_-N^+^(CH_3_)_2_-), 3.92 (s, 10H, -CH_2_-), 4.57 (m, 20H, -O-C*H*_2_-C(O)-NH-), 6.84 (s, 10H, ArH); ^13^С NMR (100 MHz, D_2_O, 298 K) δ (ppm): 18.23, 33.06, 43.13, 47.19, 51.07, 61.58, 62.89, 67.54, 114.61, 128.87, 148.91, 171.69; IR (ν/cm^−1^): 2987, 2970, 2918 (-CH_2_-, -CH=), 1662 (С=О), 1200 (Ar-O-CH_2_-), 1170, 1032 (-SO_3_^−^); ESIMS *m*/*z*: calcd for [M^+^], 3113.18, found for [M + 3Na + H]^4+^, 795.54; Anal calcd for C_125_H_210_N_20_O_50_S_10_: C, 48.22; H, 6.80; N, 9.00; S, 10.30; found: C, 49.58; H, 6.30; N, 8.83; S, 10.23.

#### 4,8,14,18,23,26,28,31,32,35-deca-[*N*-(2',2'-dimethyl-2'-(4''-sulfonatobutyl)ammoniummethyl)carbamoylmethoxy]pillar[5]arene (**4**)

Yield 0.36 g (69%). ^1^H NMR (400 MHz, D_2_O, 298 K) δ (ppm): 1.75 (m, -20H, CH_2_-CH_2_-C*H*_2_-CH_2_-SO_3_^−^), 1.75 (m, 20H, -CH_2_-C*H*_2_-СH_2_-CH_2_-SO_3_^−^), 2.62 (m, 20H, -CH_2_-CH_2_-СH_2_-C*H*_2_-SO_3_^−^), 2.91 (m, 20H, -C*H*_2_-CH_2_-СH_2_-CH_2_-SO_3_^−^), 3.11 (m, 60H, -N^+^(C*H*_3_)_2_-), 3.50 (m, 20H, -NH-CH_2_-C*H*_2_-N^+^(CH_3_)_2_-), 3.72–3.73 (m, 20H, -NH-C*H*_2_-CH_2_-N^+^(CH_3_)_2_-), 3.86 (s, 10H, -CH_2_-), 4.53 (br s, 20H, -O-C*H*_2_-C(O)-NH-), 6.84 (s, 10H, ArH); ^13^С NMR (100 MHz, D_2_O, 298 K) δ (ppm): 20.88, 21.08, 33.03, 43.36, 49.95, 56.56, 61.64, 63.83, 67.47, 128.82, 149.05, 146.77, 171.58; IR (ν/cm^−1^): 2970, 2941 (-CH_2_-, -CH=), 1656 (С=О), 1200 (Ar-O-CH_2_-), 1168, 1034 (-SO_3_^−^); ESIMS *m*/*z*: calcd for [M^+^], 3253.34; found for [M + 4Na]^4+^, 836.1; Anal cacld for calcd for C_135_H_230_N_20_O_50_S_10_: C, 49.83; H, 7.12; N, 8.61; S, 9.85; found: C, 49.63; H, 7.19; N, 8.54; S, 9.62.

### ^1^H diffusion ordered spectroscopy (DOSY)

^1^H diffusion ordered spectroscopy (DOSY) spectra were recorded on a Bruker Avance 400 spectrometer at 9.4 tesla at a resonating frequency of 400.17 MHz for ^1^H using a BBO Bruker 5 mm gradient probe. The temperature was regulated at 298 K and no spinning was applied to the NMR tube. DOSY experiments were performed using the stimulated echo (STE) bipolar gradient pulse pair (stebpgp1s) pulse sequence with 16 scans of 16 data points collected. The maximum gradient strength produced in the *z* direction was 5.35 G mm^−1^. The duration of the magnetic field pulse gradients (δ) was optimized for each diffusion time (Δ) in order to obtain a 2% residual signal with the maximum gradient strength. The values of δ and Δ were 1.800 μs and 100 ms, respectively. The pulse gradients were incremented from 2 to 95% of the maximum gradient strength in a linear ramp [49].

### Electrospray ionization mass spectrometry

Electrospray ionization mass spectra (ESI) were obtained on an AmazonX mass spectrometer (Bruker Daltonik GmbH, Bremen, Germany). The measurements were carried out in the regime of positive ions in the range of *m*/*z* from 100 to 2800. The voltage on the capillary was −4500 V. Nitrogen was used as the gas-drier with a temperature of 300 °C and a flow rate of 10 L∙min^−1^. The compounds were dissolved in acetonitrile to a concentration of 10^−6^ g/L. The data was processed using DataAnalysis 4.0 (Bruker Daltonik GmbH, Bremen, Germany).

### UV–vis spectroscopy

UV–vis spectra were recorded using the Shimadzu UV-3600 spectrometer (cell thickness 1 cm, slit width 1 nm). Deionized water with a resistivity >18.0 MΩ cm was used to prepare the solutions. The deionized water was obtained from a Millipore-Q purification system. Recording of the absorption spectra of the mixtures of pillar[5]arenes **3** and **4** (1 × 10^−5^ М) with AgNO_3_ (1 × 10^−4^ М) was carried out after mixing the solutions at 293 K.

### Dynamic light scattering (DLS)

The particle size distribution formed as a result of self-association of the pillar[5]arenes **2**–**4** was determined at 20 °С by dynamic light scattering using a nanoparticle size analyzer (Zetasizer Nano ZS, Malvern) in quartz cuvettes. The instrument contains a 4 mW He–Ne laser operating at a wavelength of 633 nm and incorporates noninvasive backscatter optics (NIBS). The measurements were performed at the detection angle of 173° and the software automatically determined the measurement position within the quartz cuvette. The experiments were carried out for each solution in triplicate. The experiments were carried out in deionized H_2_O. The concentration of the compounds was 10^−3^–10^−5^ M and the macrocycle/AgNO_3_ systems with a ratio of 2:1, 1:1, 1: 5, 1:10, 1:15 in the concentration range of 10^−3^–10^−5^ M were tested. The solutions were held for 1 h and then the particle size was measured.

### Transmission electron microscopy (TEM)

Transmission electron microscopy (TEM) analysis of **3**/Ag^+^ and **4**/Ag^+^ associates was carried out using the Hitachi HT7700 Exalens transmission electron microscope with an Oxford Instruments X-Max^n^ 80T EDS detector. For the sample preparation, 10 μL of the suspension was placed on the Formvar™/carbon coated 3 mm copper grid, which was then dried at room temperature. After complete drying, the grid was placed into the transmission electron microscope using a special holder for microanalysis. The analysis was held at the accelerating voltage of 80 kV in scanning TEM mode using an Oxford Instruments X-Max^n^ 80T EDS detector.

### In vitro cell viability

The characterization of the A549 cell viability changes under the pillar[5]arenes **3** and **4** action was performed by the MTT assay. A cell suspension of 150 μL/well was seeded in a 96-well plate at a final concentration of 1 × 10^4^ cells/well. After 24 h the media from the wells was aspirated and replaced by fresh supplemented test compounds in a volume of 100 μL. After 24 h of incubation in the presence of pillar[5]arenes **3** and **4**, 10 μL of MTT solution (5 mg/mL) was added to the wells. The plates were incubated at 37 °C for 4 h. Then the medium was aspirated from the wells and 100 μL of DMSO (to dissolve the crystals) was added to each well. The probes were incubated at 37 °C for 5 min. The absorbance was measured at 570 nm using a xMark spectrophotometer (BIO-RAD, USA). Mathematical processing of the results was carried out using the nonparametric Cramer–Welch criterion as a reliability criterion. *p* ≤ 0.05 was taken as a reliable level of significance. The calculation of the standard deviation of the results of the experiments was carried out in using MS-Excel. To determine the cytotoxicity of the complexes with **3**/Ag^+^ and **4**/Ag^+^ and to obtain a stable mixture of compounds, solutions **3** or **4** and AgNO_3_ in distilled water was mixed in a molar ratio of 1:10 and incubated for 30 min at 37 °C. The IC_50_ value using the data obtained in the MTT assay was determined. A series of concentrations was tested (from 5 μg/mL to 0.1 μg/mL). Primary data processing was performed in Microsoft Excel, after which the average values and silver concentrations were loaded into the calculator [50] to calculate the half maximum inhibitory concentration.

## Supporting Information

File 1 Additional experimental parameters and results.
