# Supplementary material for: Nanoparticles based on the zwitterionic pillar[5]arene and Ag+: synthesis, self-assembly and cytotoxicity in the human lung cancer cell line A549
Source: Beilstein J Nanotechnol. 2020 Mar 5;11:421–31. doi: 10.3762/bjnano.11.33 (PMC7082700; doi:10.3762/bjnano.11.33)
Supplement: File 1 — Additional experimental parameters and results. [file Beilstein_J_Nanotechnol-11-421-s001.pdf]

BEILSTEIN JOURNAL OF NANOTECHNOLOGY

Supporting Information

for

Nanoparticles based on the zwitterionic pillar[5]arene and Ag^+: synthesis, self-assembly and cytotoxicity in the human lung cancer cell line A549

Dmitriy N. Shurpik, Denis A. Sevastyanov, Pavel V. Zelenikhin, Pavel L. Padnya, Vladimir G. Evtugyn, Yuriy N. Osin and Ivan I. Stoikov

Beilstein J. Nanotechnol. **2020,** *11,* 421–431. doi:10.3762/bjnano.11.33

Additional experimental parameters and results

License and Terms: This is a supporting information file under the terms of the Creative Commons Attribution License (https://creativecommons.org/licenses/by/4.0). Please note that the reuse, redistribution and reproduction in particular requires that the authors and source are credited.
The license is subject to the *Beilstein Journal of Nanotechnology* terms and conditions: (https://www.beilstein-journals.org/bjnano)

1. NMR, MALDI TOF MS, ESI MS, IR spectra of compounds 2-4. .... **S2**

2. UV spectra **S8**

3. Dynamic light scattering. **S8**

4. TEM images. **S10**

5. The 2D ^{1}H-^{1}H NOESY, ROESY NMR spectra. **S13**

6. Diffusion experiments. **S15**

1. NMR, MALDI TOF MS, ESI MS, IR spectra of compounds 2–4.

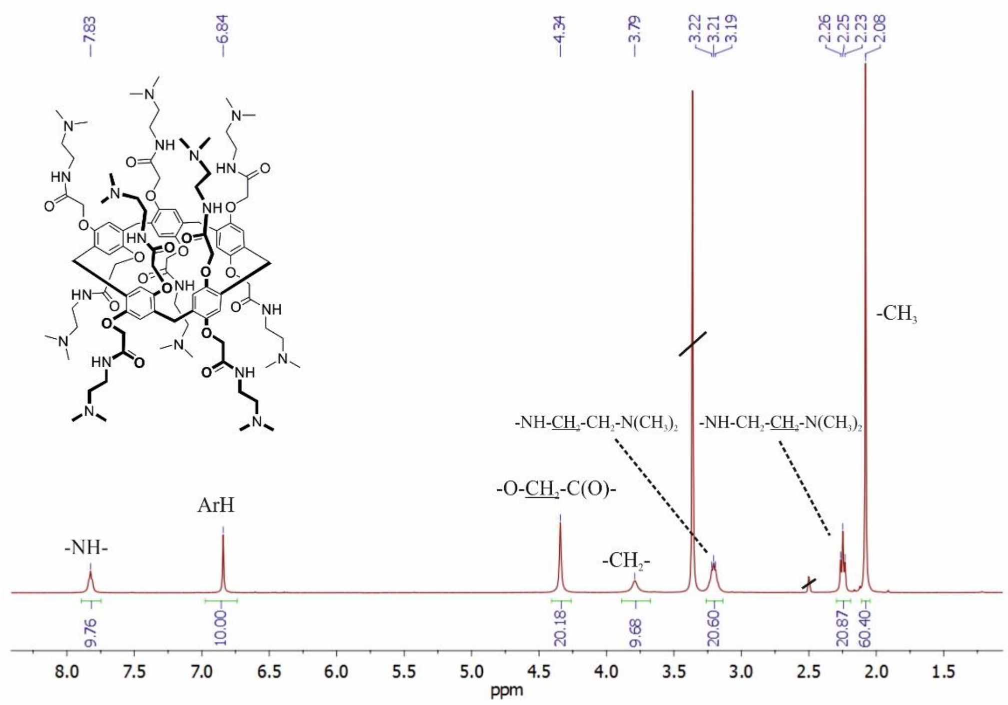

Figure S1: ^{1}H NMR spectrum of 4,8,14,18,23,26,28,31,32,35-deca[*N*-(2’,2’-dimethylaminoethyl)carbamoylmethoxy]pillar[5]arene (**2**), DMSO-d_6, 298 K, 400 MHz.

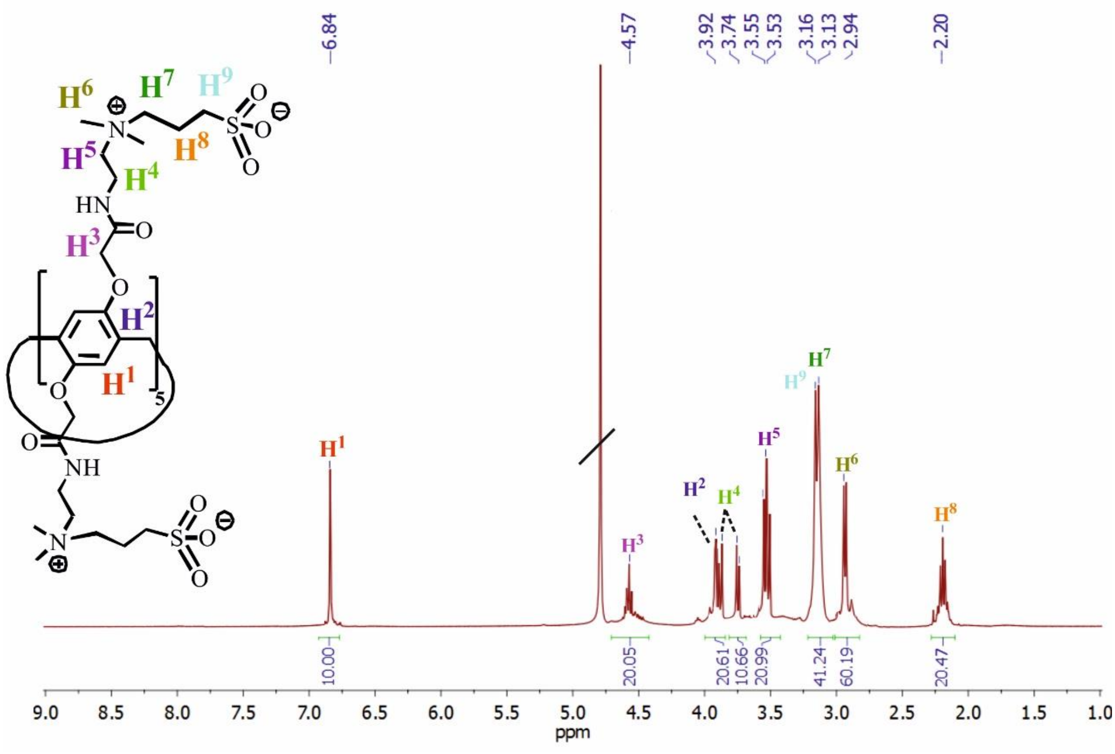

Figure S2: ^{1}H NMR spectrum of 4,8,14,18,23,26,28,31,32,35-deca-[*N*-(2',2'-dimethyl-2'-(3''-sulfonatopropyl)ammoniummethyl)carbamoylmethoxy]pillar[5]arene (**3**), D$_2$O, 298 K, 400 MHz.

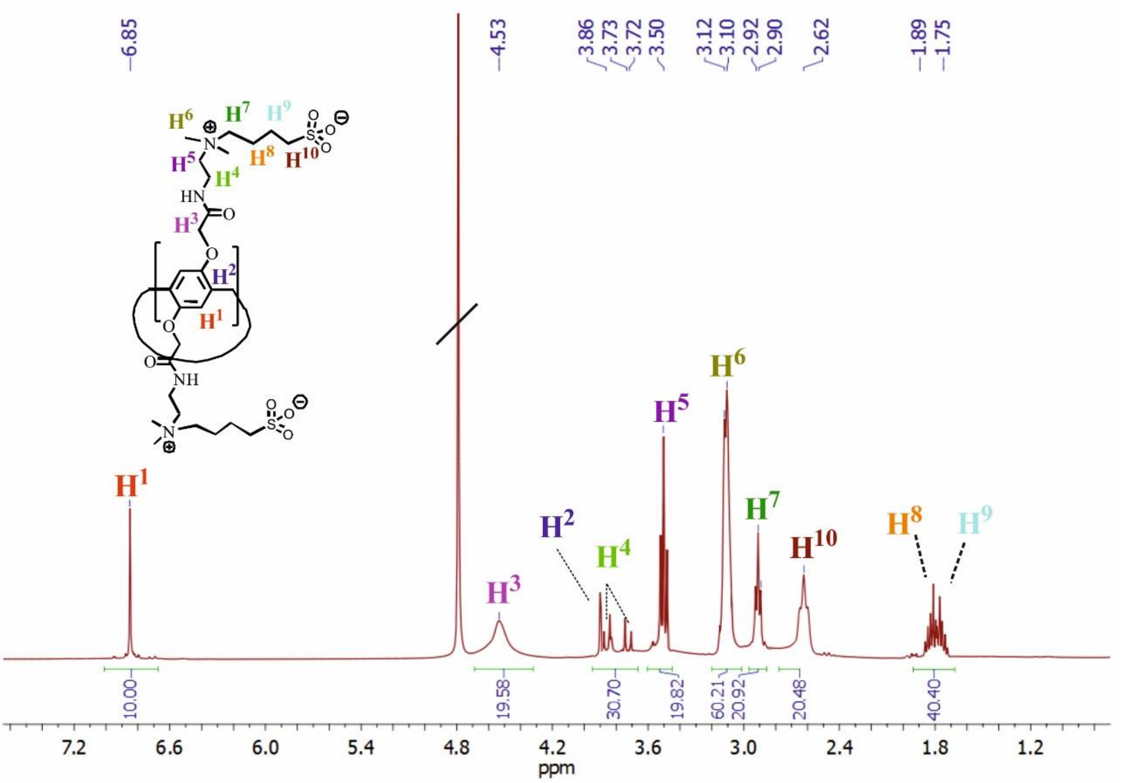

Figure S3: ^{1}H NMR spectrum of 4,8,14,18,23,26,28,31,32,35-deca[*N*-(2',2'-dimethyl-2'-(4"-sulfonatobutyl)ammoniummethyl)carbamoylmethoxy]pillar[5]arene (**4**), D_2O, 298 K, 400 MHz.

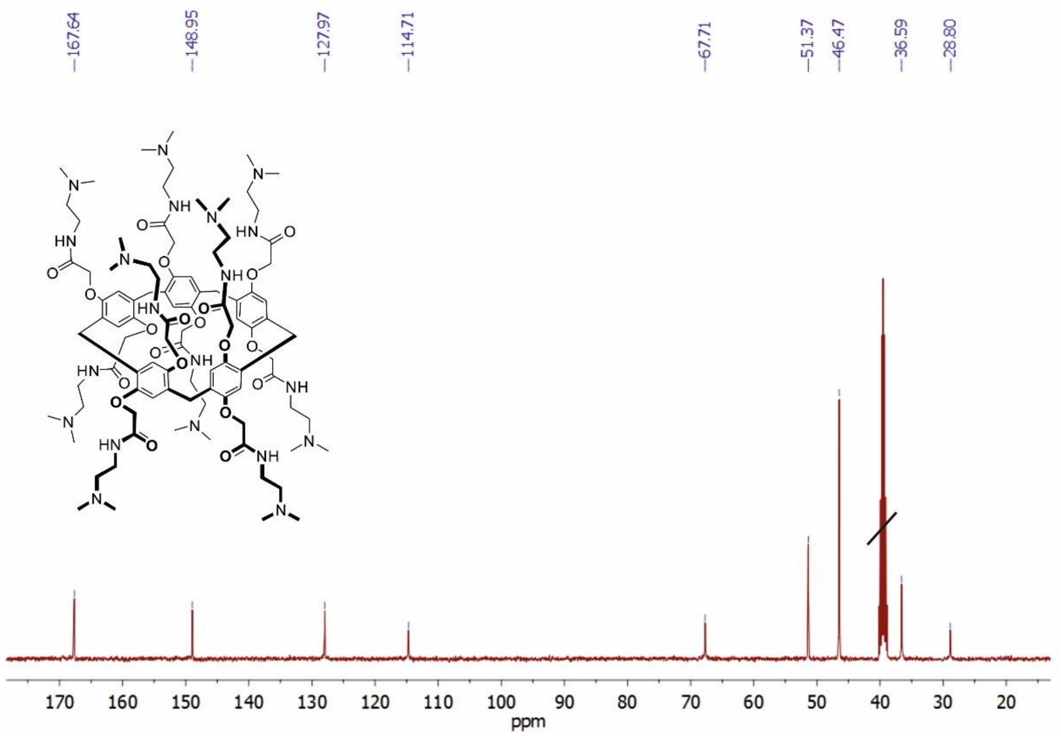

Figure S4: ^{13}C NMR spectrum of 4,8,14,18,23,26,28,31,32,35-deca[*N*-(2',2'-dimethylaminoethyl)carbamoylmethoxy]pillar[5]arene (**2**), DMSO-d_6, 298 K, 100 MHz.

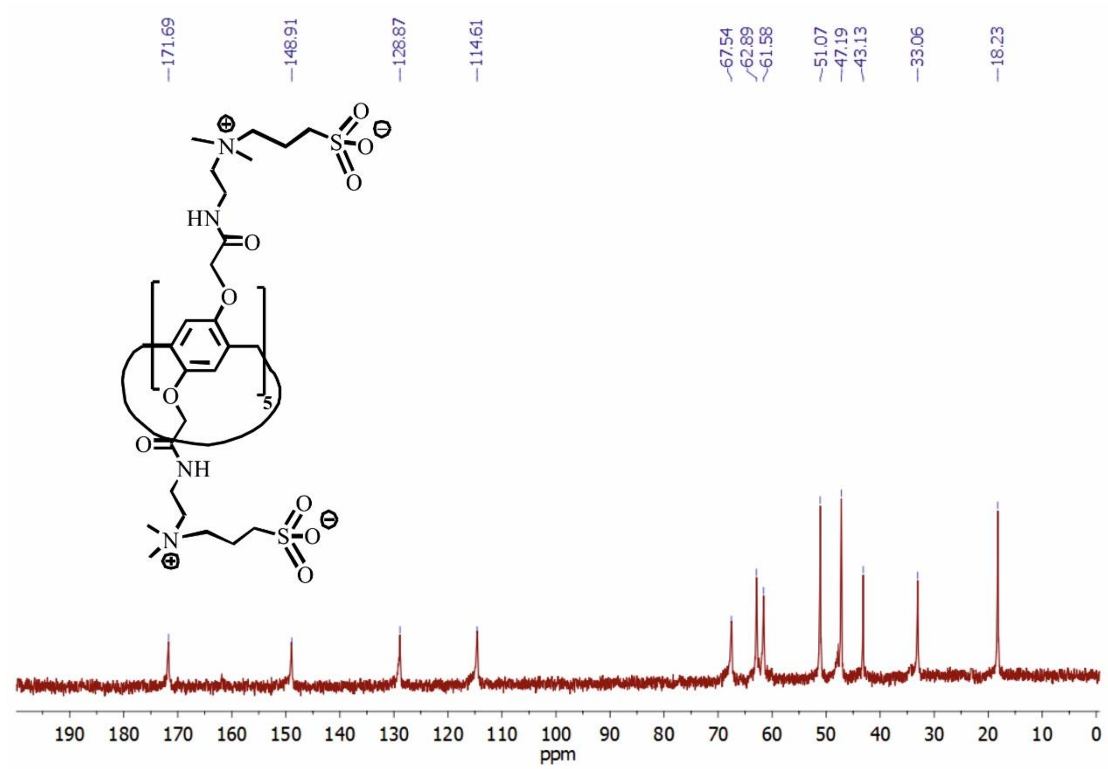

Figure S5: ^{13}C NMR spectrum of 4,8,14,18,23,26,28,31,32,35-deca[*N*-(2',2'-dimethyl-2'-(3''-sulfonatopropyl)ammoniummethyl)carbamoylmethoxy]pillar[5]arene (**3**), D_2O, 298 K, 100 MHz.

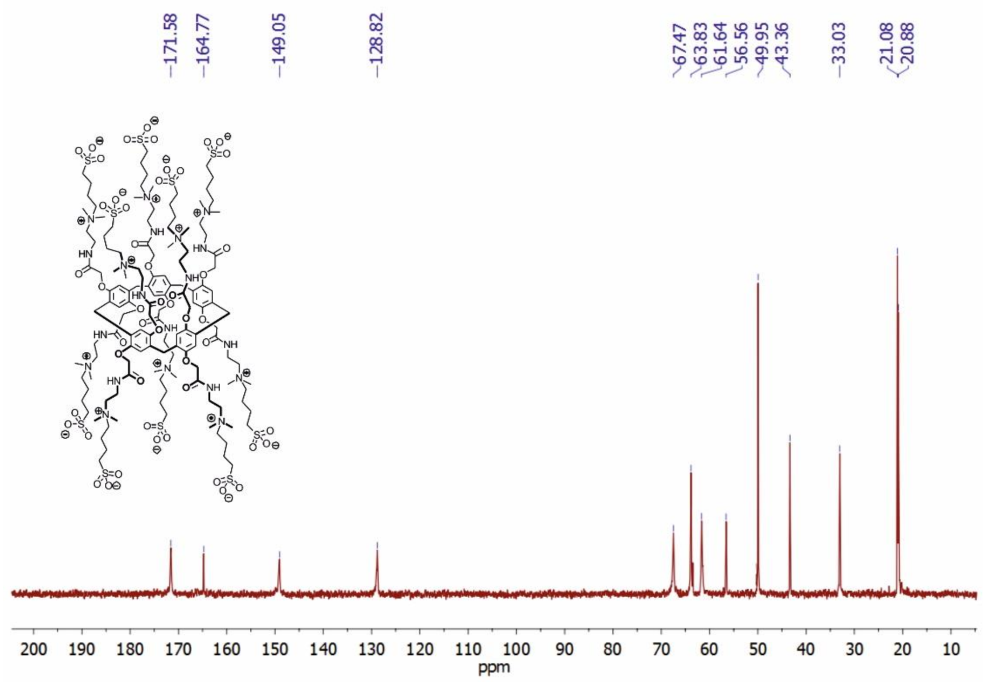

Figure S6: ^{13}C NMR spectrum of 4,8,14,18,23,26,28,31,32,35-deca[*N*-(2',2'-dimethyl-2'-(4''-sulfonatobutyl)ammoniummethyl)carbamoylmethoxy]pillar[5]arene (**4**), D_2O, 298 K, 100 MHz.

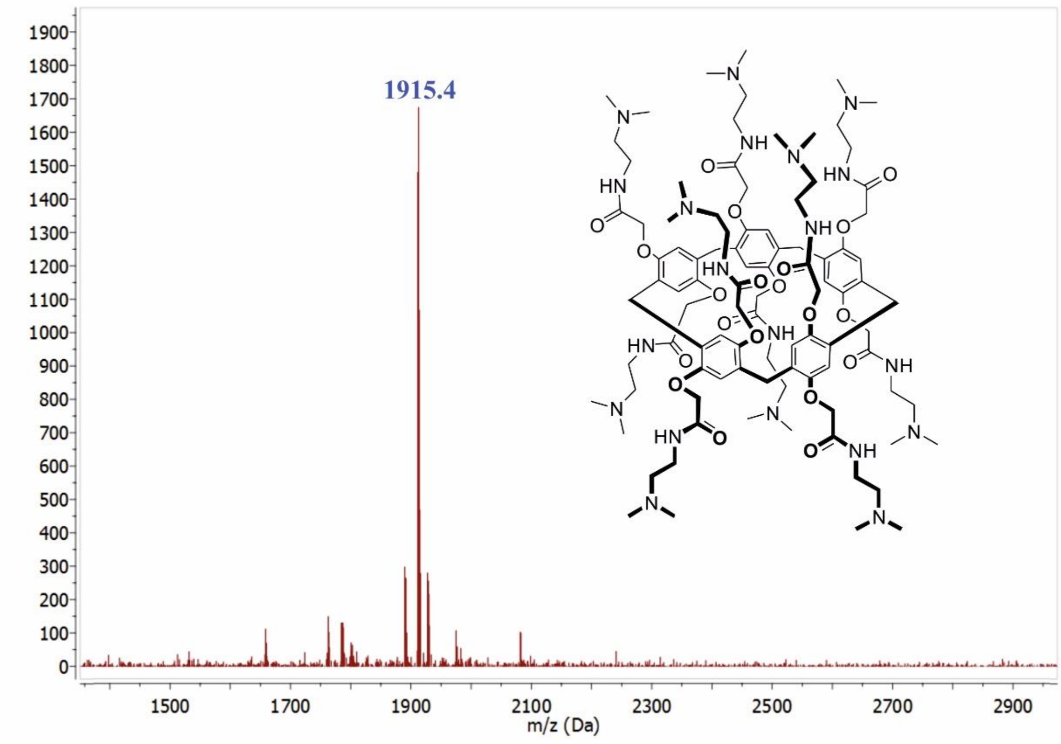

Figure S7: Mass spectrum (MALDI–TOF, 4-nitroaniline matrix) of 4,8,14,18,23,26,28,31,32,35-deca[*N*-(2’,2’-dimethylaminoethyl)carbamoylmethoxy]-pillar[5]arene (**2**).

Acquisition Parameter

Ion Source Type	ESI	Ion Polarity	Positive	Alternating Ion Polarity	off
Mass Range Mode	UltraScan	Scan Begin	70 m/z	Scan End	2700 m/z
Capillary Exit	140.0 V	n/a	n/a	Trap Drive	73.0
Accumulation Time	486 µs	Averages	5 Spectra	Auto MS/MS	off

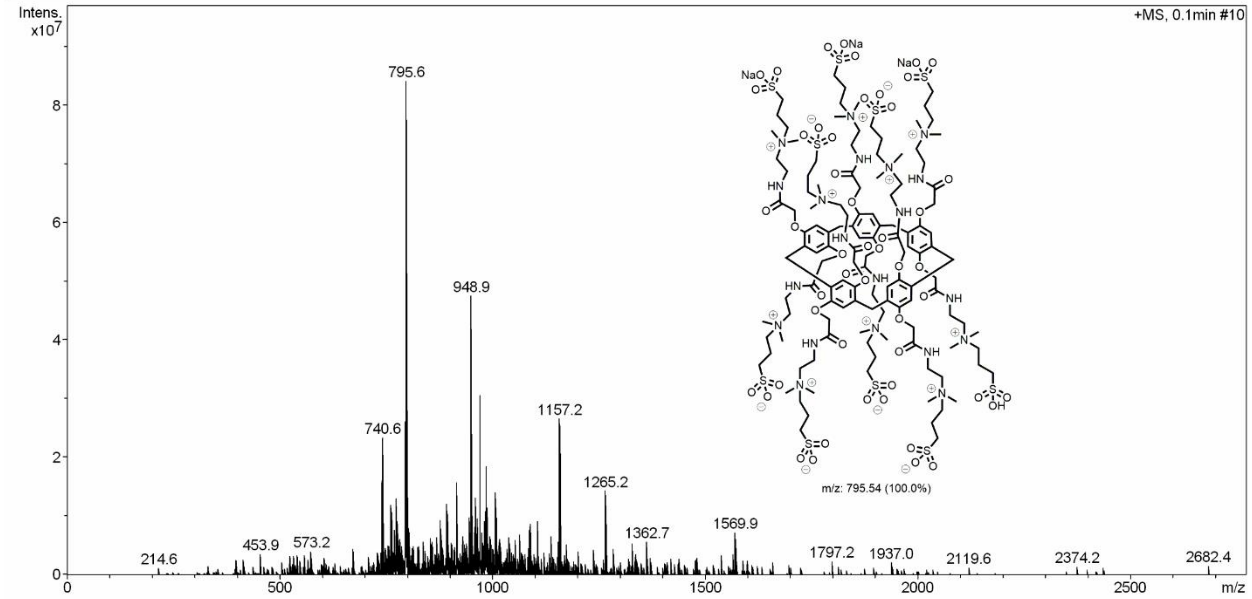

Figure S8: Mass spectrum (ESI) of 4,8,14,18,23,26,28,31,32,35-deca[*N*-(2',2'-dimethyl-2'-(3''-sulfonatopropyl)ammoniummethyl)carbamoylmethoxy]pillar[5]arene (**3**).

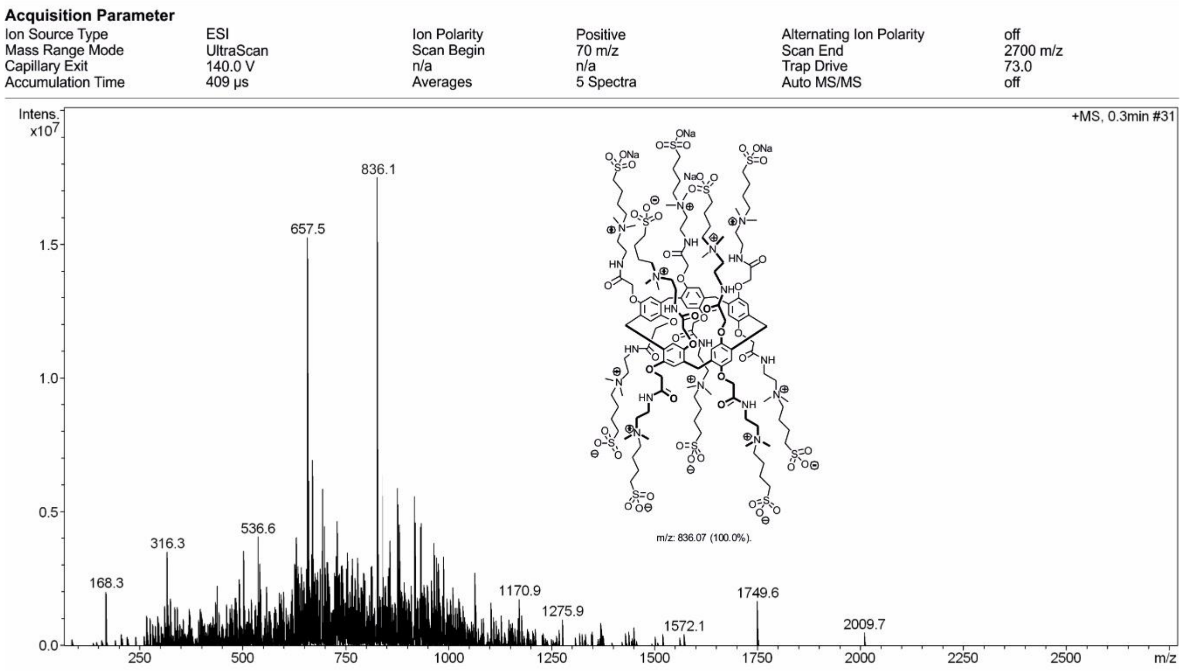

Figure S9: Mass spectrum (ESI) of 4,8,14,18,23,26,28,31,32,35-deca[*N*-(2',2'-dimethyl-2'-(4''-sulfonatobutyl)ammoniummethyl)carbamoylmethoxy]pillar[5]arene (**4**).

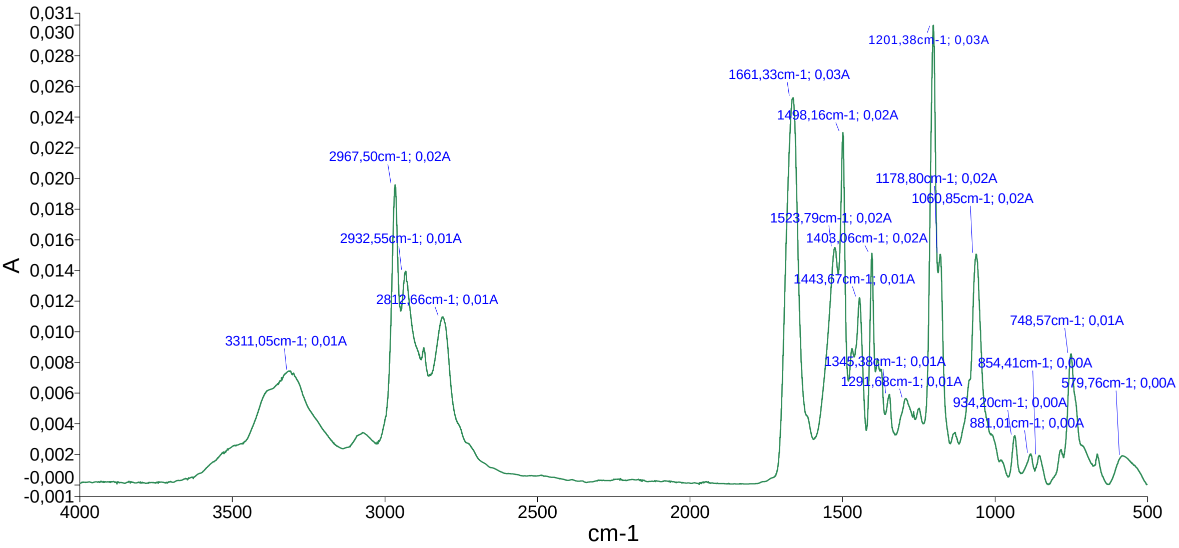

Figure S10: IR spectrum of 44,8,14,18,23,26,28,31,32,35-deca[*N*-(2’,2’-dimethylaminoethyl)-carbamoylmethoxy]pillar[5]arene (**2**).

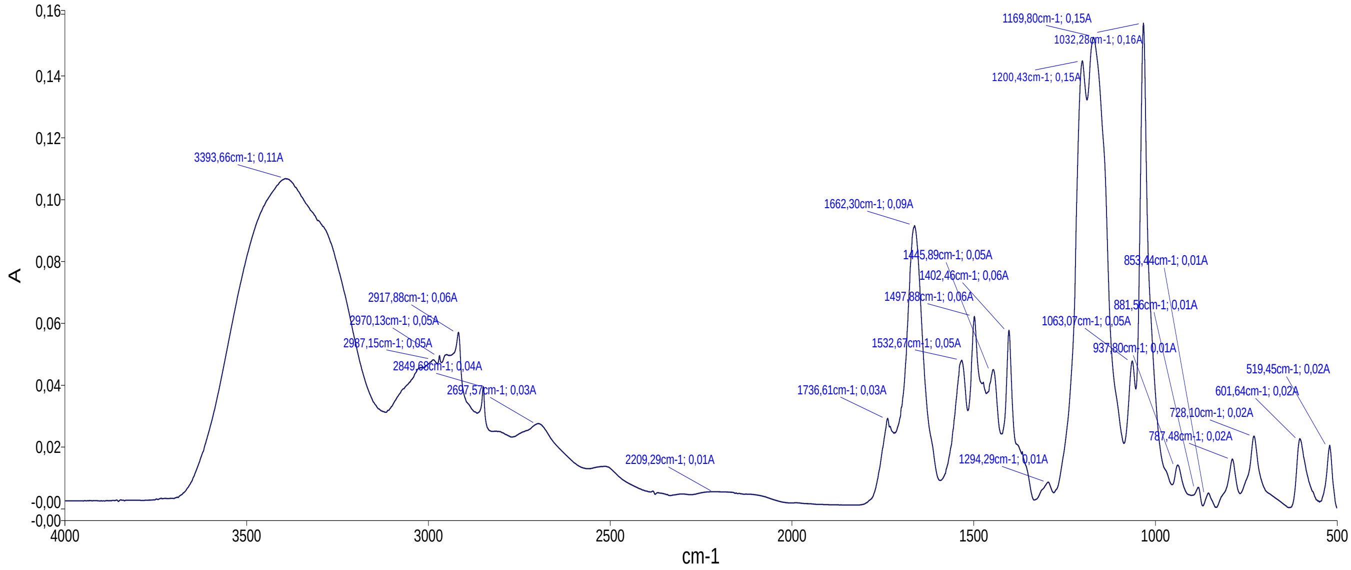

Figure S11: IR spectrum of 4,8,14,18,23,26,28,31,32,35-deca[*N*-(2',2'-dimethyl-2'-(3''-sulfonatopropyl)ammoniummethyl)carbamoylmethoxy]pillar[5]arene (**3**).

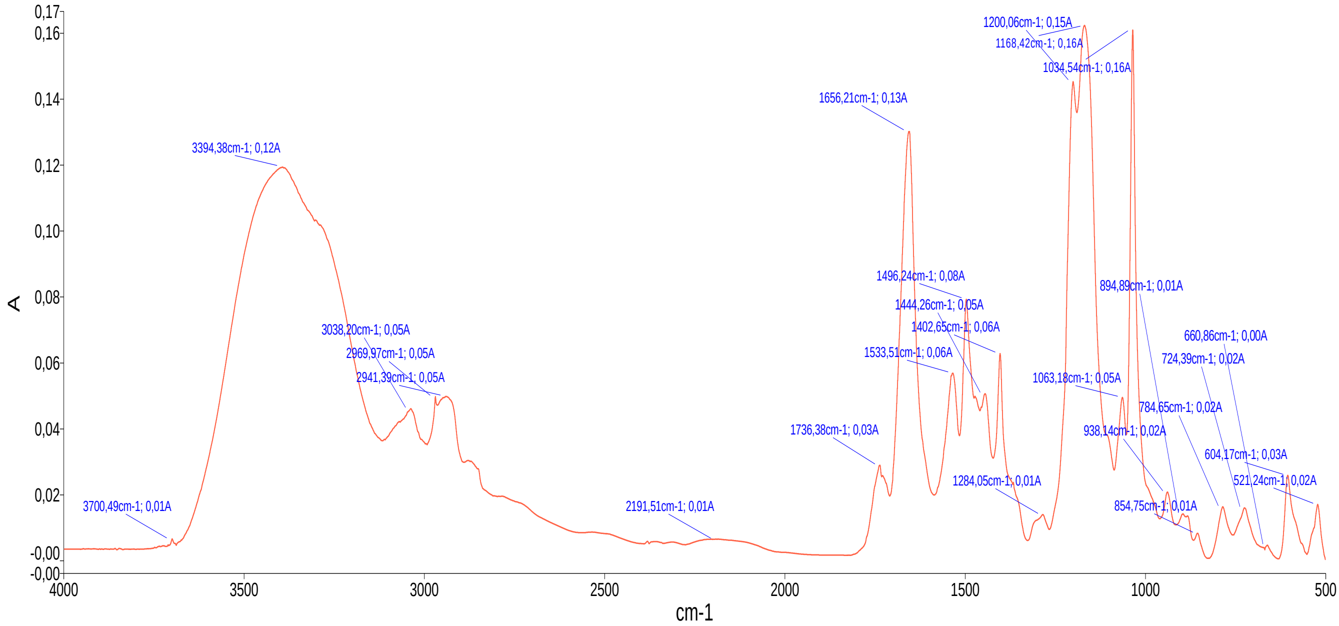

Figure S12: IR spectrum of 4,8,14,18,23,26,28,31,32,35-deca[*N*-(2',2'-dimethyl-2'-(4''-sulfonatobutyl)ammoniummethyl)carbamoylmethoxy]pillar[5]arene (**4**).

2. UV spectra.

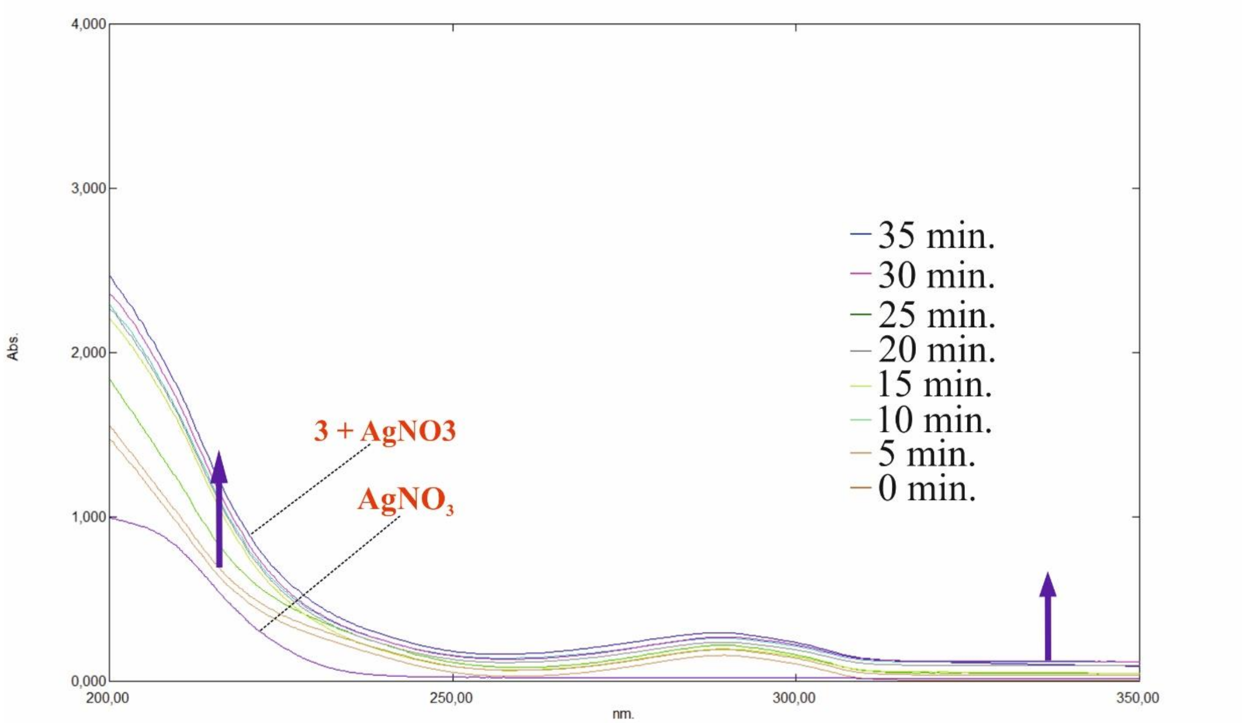

Figure S13: UV-vis spectra of pillar[5]arene 3 (10^{-5} M) with $AgNO_3$ (10^{-4} M) in water with the time interval 0–35 min.

3. Dynamic light scattering.

Table S1. The average hydrodynamic diameter and polydispersity index (PDI) of associates **3**/**Ag**$^+$ in various ratios.

Ratio of reagents / Concentration	Size of associate **3**/**Ag**$^+$, nm (PDI)				
	2:1	1:1	1:5	1:10	1:15
10^{-3}	294.0±32.2 (0.51)	105.9±4.7 (0.34)	116.7±5.3 (0.41)	75.4±0.9 (0.13)	142.2±7.9 (0.22)
10^{-4}	111.5±3.2 (0.23)	151.5±3.2 (0.22)	160.7±3.1 (0.17)	122.12±0.001 (0.041)	675.5±195.3 (0.79)
10^{-5}	115.9±5.1 (0.36)	155.6±2.7 (0.24)	101.9±0.7 (0.12)	142.7±3.4 (0.22)	630.2±152.2 (1)

Table S2. The average hydrodynamic diameter and polydispersity index (PDI) of associates **4**/**Ag**$^{+}$ in various ratios.

Ratio of reagents / Concentration	Size of associate **4**/**Ag**$^{+}$, nm (PDI)				
	2:1	1:1	1:5	1:10	1:15
10^{-3}	60.4±2.1 (0.40)	82.7±4.3 (0.45)	108.3±4.2 (0.34)	78.7±2.4 (0.31)	75.0±5.3 (0.48)
10^{-4}	83.9±25.3 (0.50)	287.5±30.8 (0.52)	203.8±38.8 (0.38)	246.6±45.32 (0.65)	244.2±89.3 (0.57)
10^{-5}	>800 (1)	>800 (1)	321.1±93.4 (0.49)	>800 (1)	773.2±112.2 (0.88)

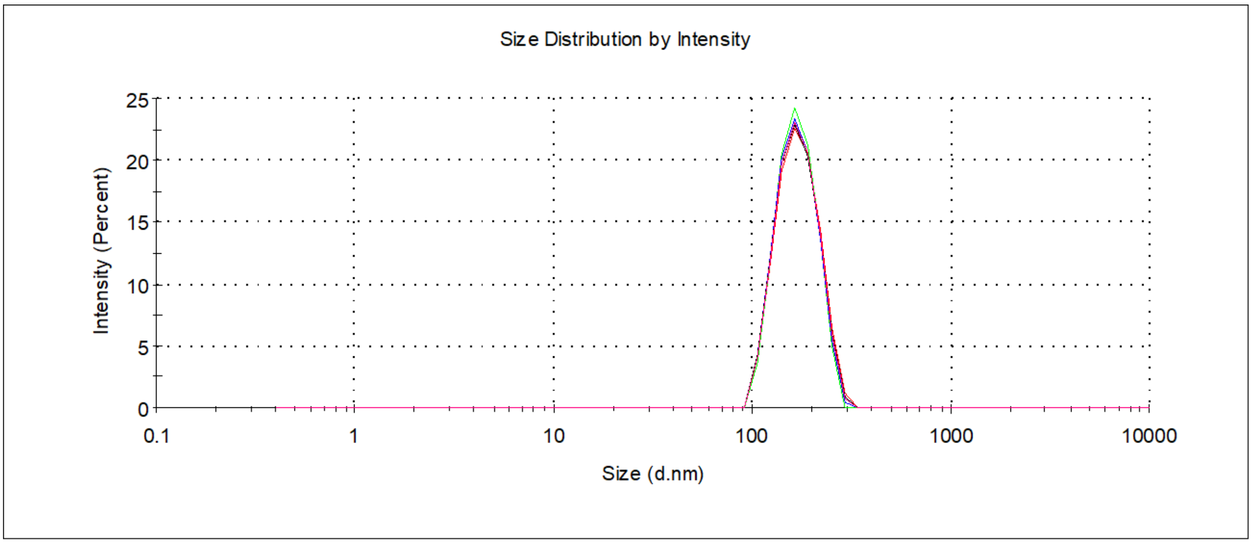

Figure S14: Size distribution of the particles by intensity for **3**/Ag^{+} (c(**3**) = 10^{-4} M, c($AgNO_3$) = 10^{-3} M, 1:10) in water.

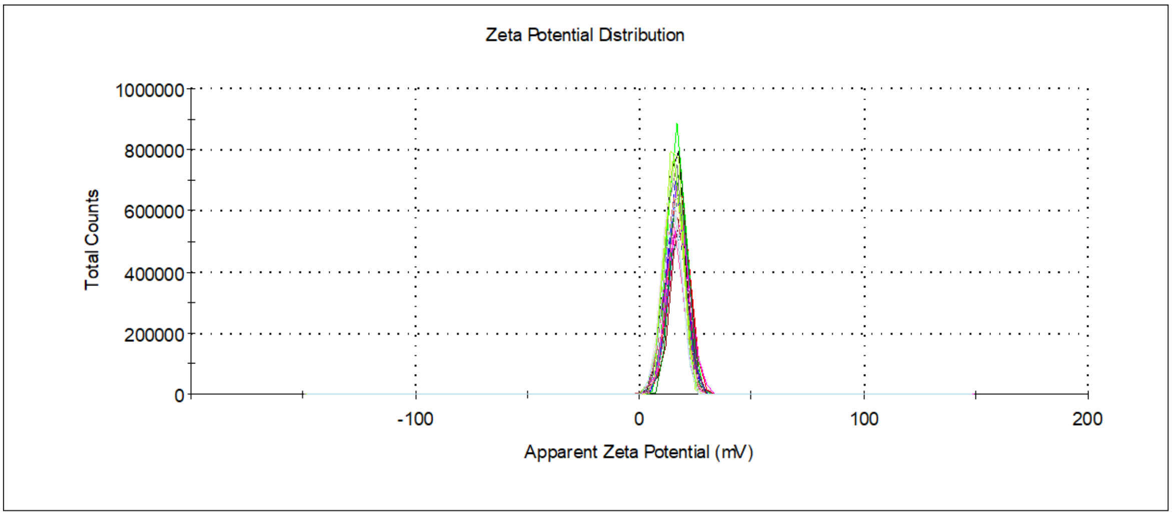

Figure S15: ζ-potential of the system **3**/Ag^+ = 1:10 (c(3) = 10^{-4} M, c($AgNO_3$) = 10^{-3} M) in water.

4. TEM images.

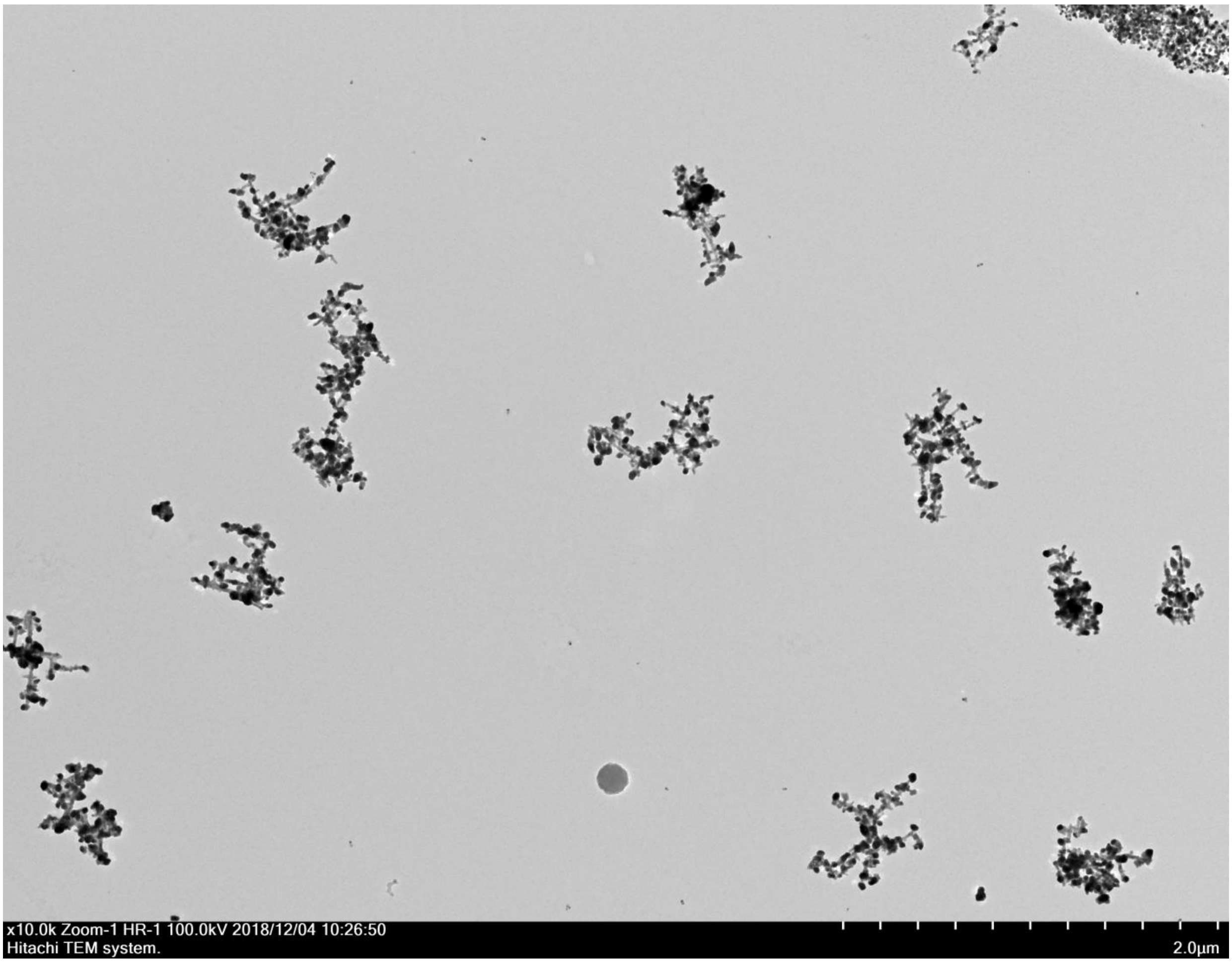

Figure S16: TEM images of pillar[5]arene **3**/Ag^+ = 1:1 associates in water (1 × 10^{-4} M).

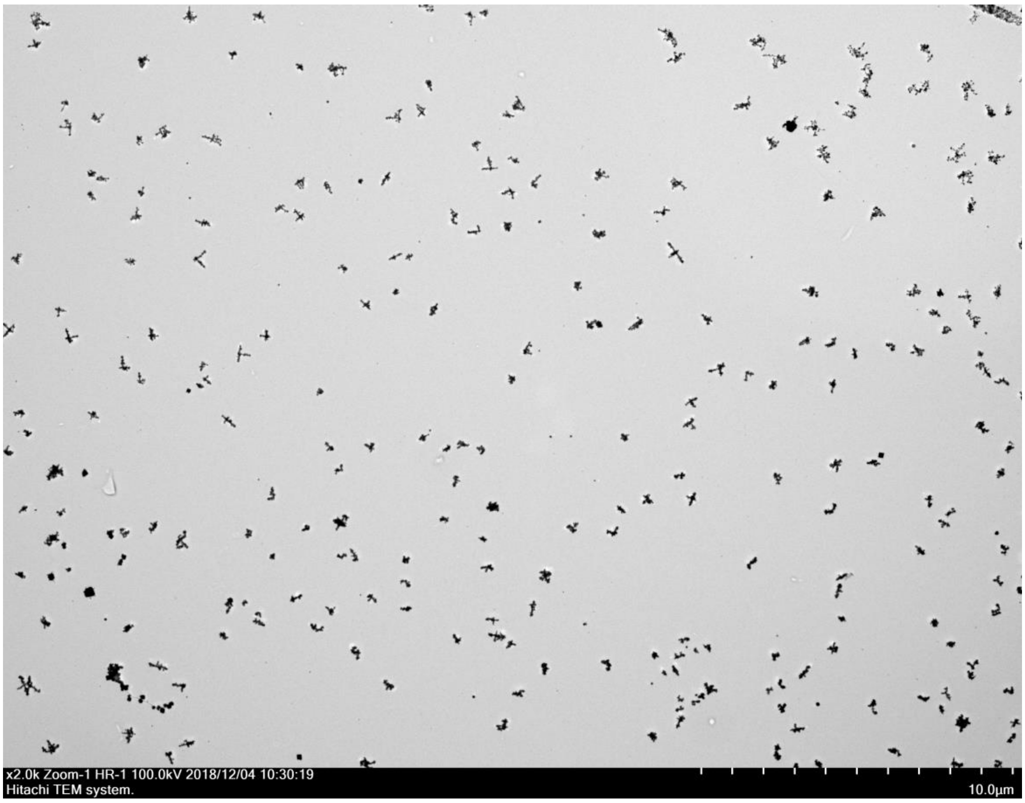

Figure S17: TEM images of pillar[5]arene **3**/Ag^+ = 1:1 associates in water (1×10^{-4} M).

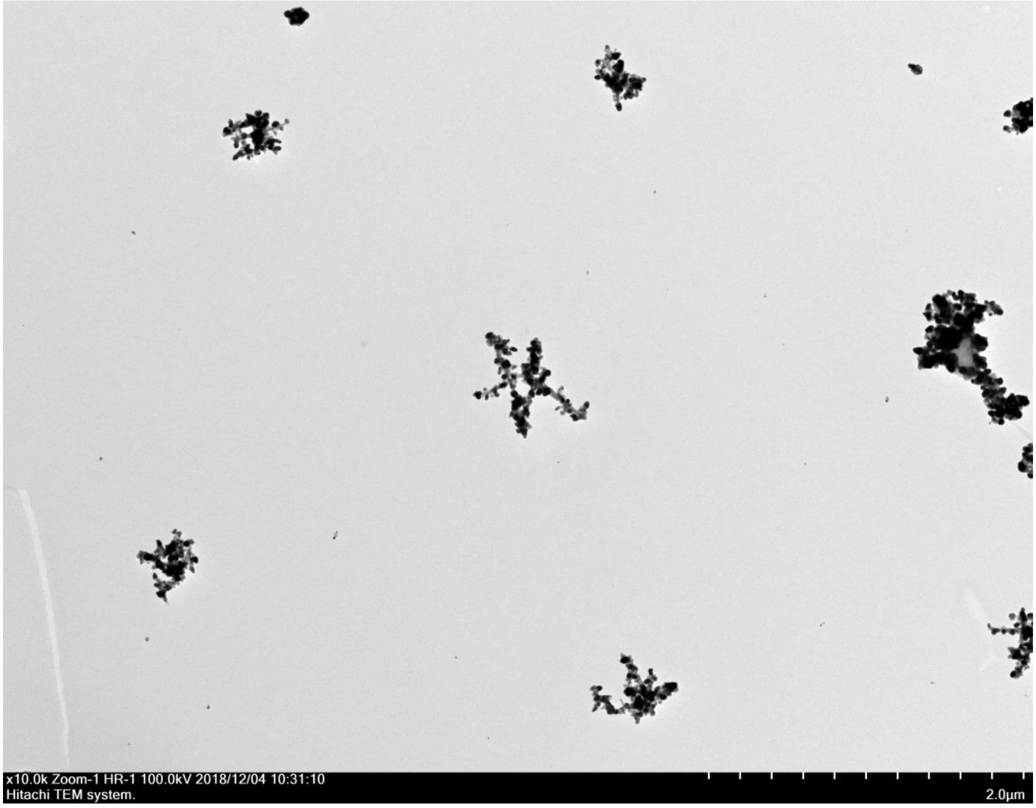

Figure S18: TEM images of pillar[5]arene **3**/Ag^+ = 1:1 associates in water (1×10^{-4} M).

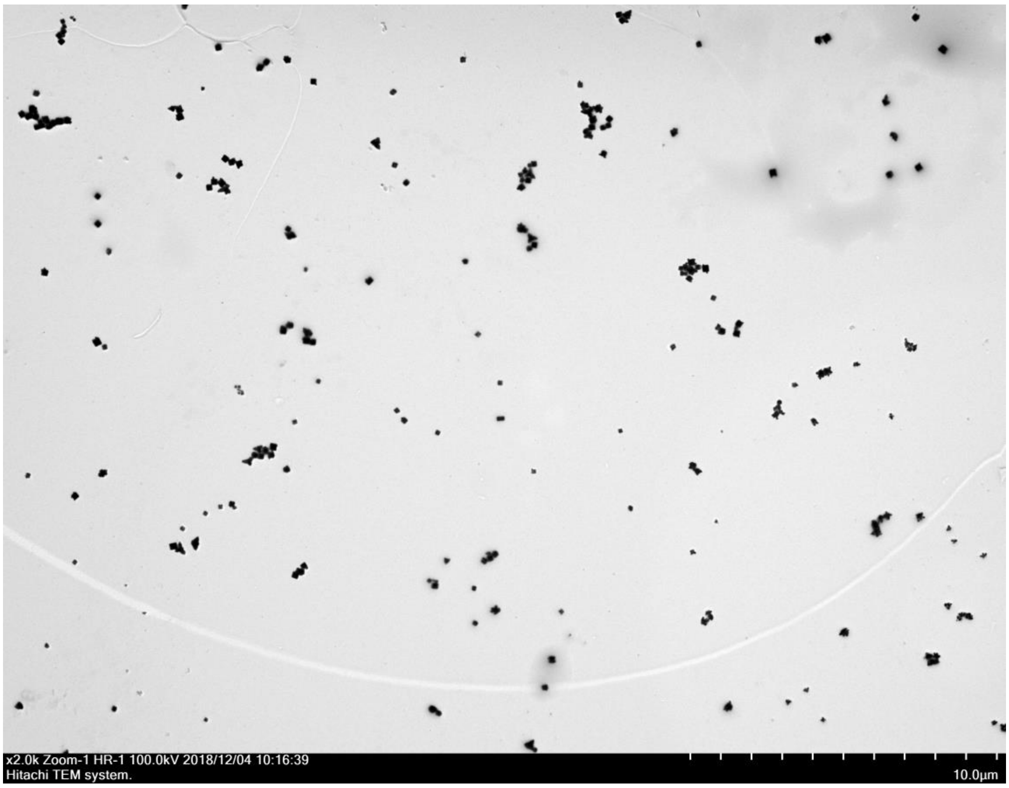

Figure S19: TEM images of pillar[5]arene **3**/Ag^+ = 1:10 associates in water (c(3) = 10^{-4} M, c($AgNO_3$) = 10^{-3} M).

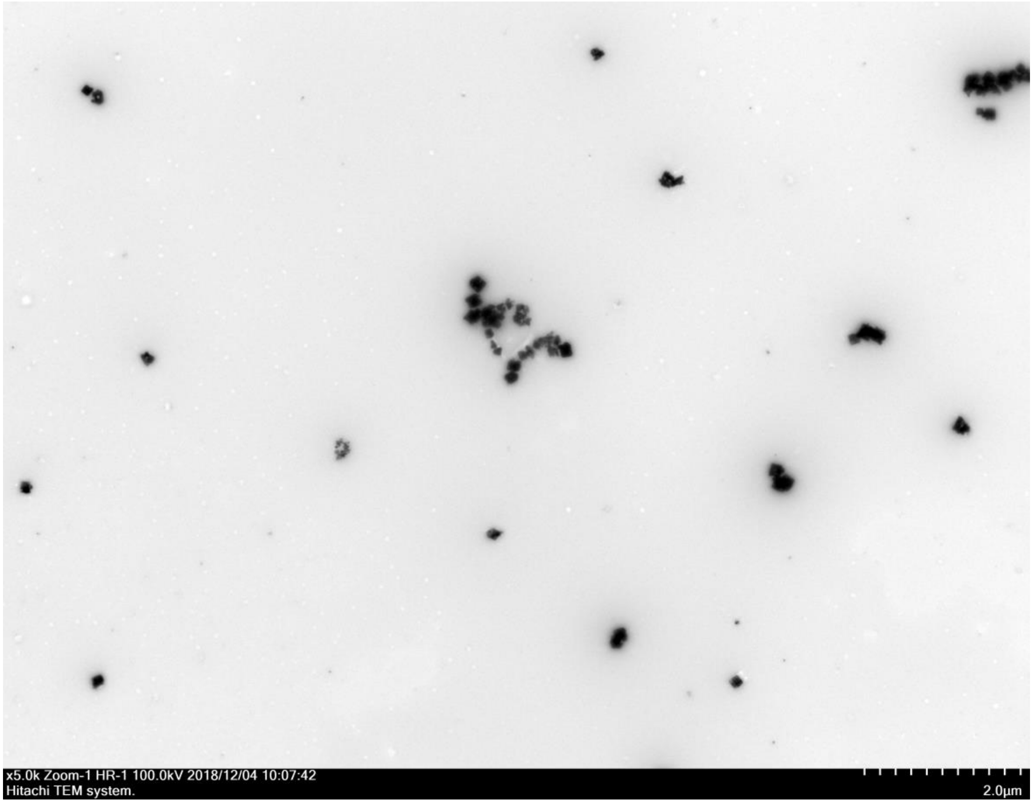

Figure S20: TEM images of pillar[5]arene **3**/Ag^+ = 1:10 associates in water (c(3) = 10^{-4} M, c($AgNO_3$) = 10^{-3} M).

5. The 2D ^{1}H-^{1}H NOESY, ROESY NMR spectra.

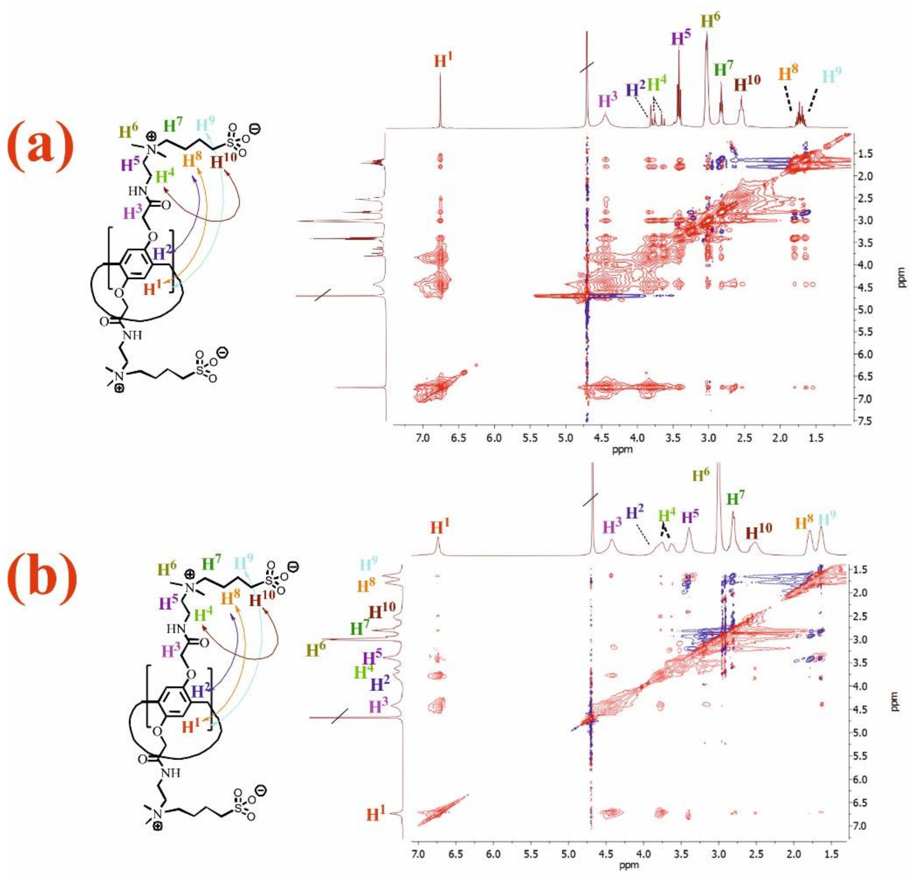

Figure S21: (a) macrocycle **4** (10^{-3} M), (b) associate **4**/Ag^{+} (1:10) in D_2O at 25 °C.

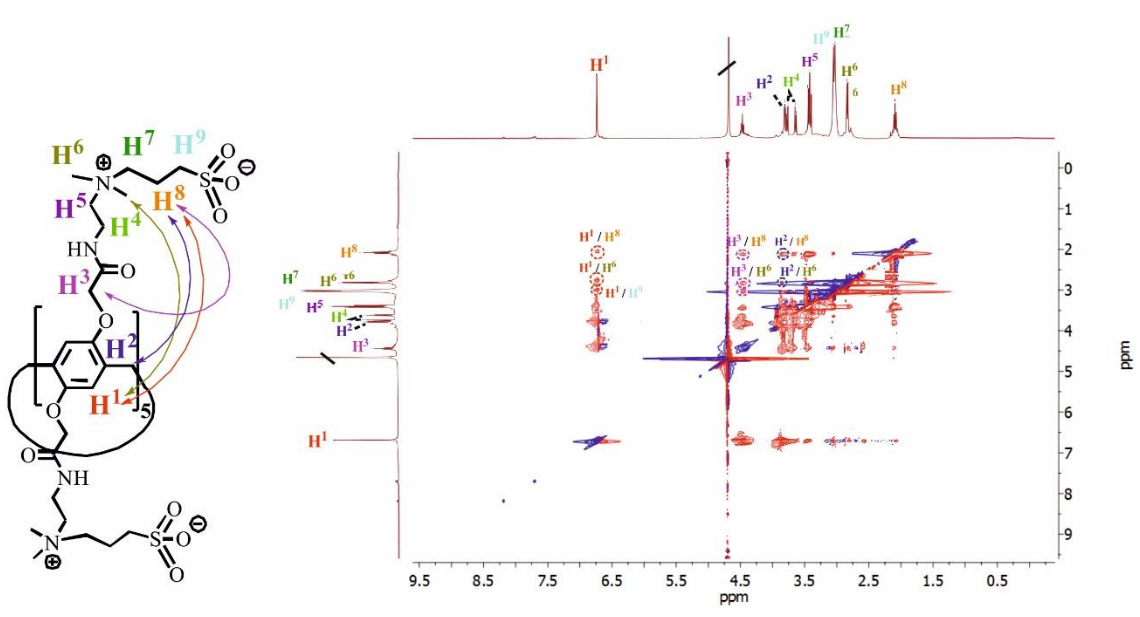

Figure S22: The 2D ^{1}H-^{1}H ROESY NMR spectra of macrocycle **3** in D_2O at 25 °C.

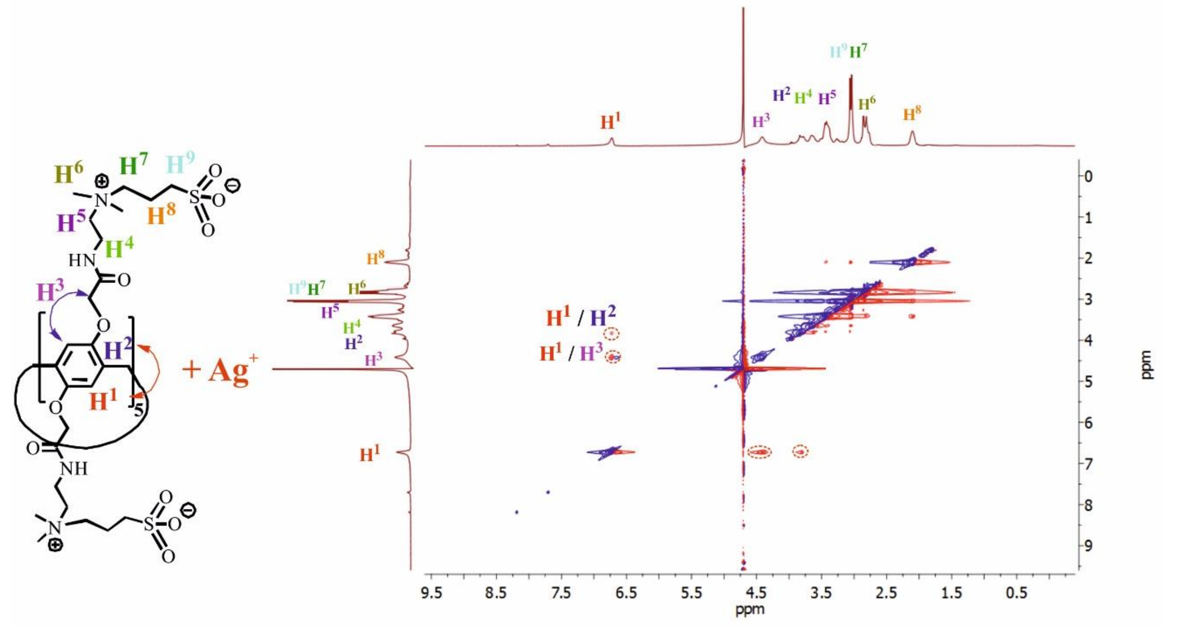

Figure S23: The 2D ^{1}H-^{1}H ROESY NMR spectra of associate **3**/Ag^+ (1:10) in D_2O at 25 °C.

6. Diffusion experiments.

Table S3. Diffusion coefficients of pure **3** and **3**/Ag^+ complex in D_2O (400 MHz, 298 K).

**C(Ag^+)/ C(3)**	**0**	**0.5**	**1**	**2**	**3**	**5**	**7**	**10**
**D, 10^{-10} m^2 s^{-1}**	**4.22**	**3.75**	**3.19**	**3.21**	**3.23**	**3.25**	**3.31**	**3.40**
**r, nm**	**0.45**	**0.6**	**0.91**	**0.82**	**0.80**	**0.79**	**0.75**	**0.70**

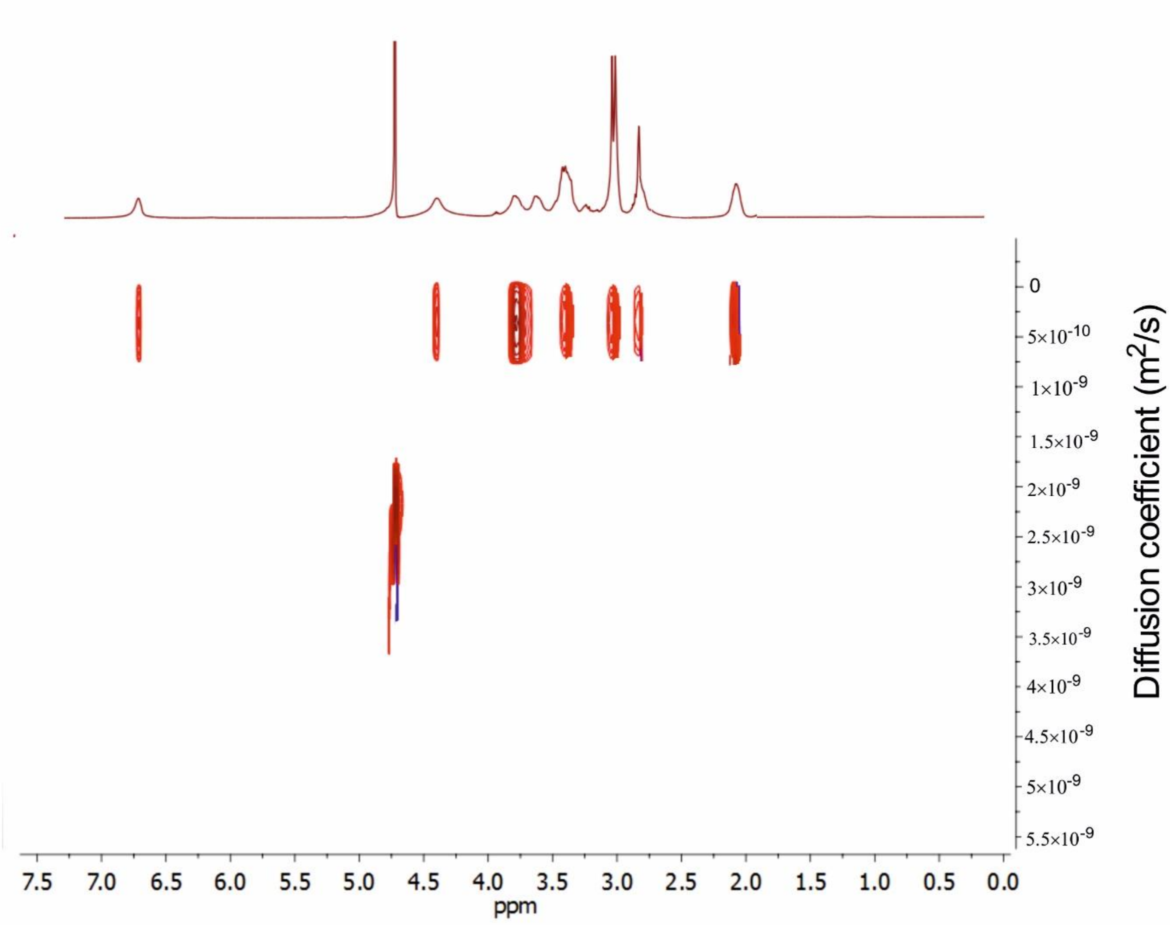

Figure S24: The DOSY spectrum **3**/Ag^+ complex in D_2O (400 MHz, 298 K).
